# Galloanseran cranial development highlights exceptions to von Baer’s laws

**DOI:** 10.1186/s13227-025-00253-7

**Published:** 2025-10-06

**Authors:** Bassel Arnaout, Kaylen Brzezinski, Albert Chen, Benjamin Steventon, Daniel J. Field

**Affiliations:** 1 Department of Earth Sciences, University of Cambridge, Cambridge, UK; 2 Department of Genetics, University of Cambridge, Cambridge, UK; 3 Department of Biology, Carleton University, Ottawa, ON Canada; 4 Museum of Zoology, University of Cambridge, Cambridge, UK

**Keywords:** Anseriformes, Galliformes, Skull, Craniofacial development, Hourglass model, von Baer’s laws

## Abstract

The remarkable morphological disparity of the animal kingdom is underpinned by changes in embryonic development across the tree of life; as such, deciphering evolutionary patterns of developmental divergence depends on investigations of different species across a range of comparable developmental stages. Among the most influential ideas regarding such developmental divergences are von Baer’s Laws of Development and Haeckel’s Theory of Recapitulation. Here, we assess several predictions following from these ideas at the tissue-level by comparing skull osteogenesis in representatives of the bird clade Galloanserae. We investigated high-resolution µCT scans of embryonic series for four galloanseran species: chickens and quails, representing Galliformes (landfowl), and ducks and geese, representing Anseriformes (waterfowl). To compare skull osteogenesis across our taxon sample, we devised a skull-specific staging system based on ossification sequences to discretise the process into five stages. During skull osteogenesis, we found that the location of the onset of ossification within each element and the direction of ossification progression were the same in all species in our sample, implying a conserved developmental programme for induction and ossification progression across Galloanserae. Moreover, we found that the appearance of synapomorphies diagnostic of broader clades often overlapped with species-specific ones during osteogenesis. Indeed, many diagnostic features of deep clades, such as osteological synapomorphies of the phylogenetically inclusive clade Galloanserae, appear at surprisingly late stages of development. These observations fail to support several predictions of von Baer’s Laws of Development and Haeckel’s Theory of Recapitulation, instead suggesting what we term a ‘braiding’ pattern of developmental divergence in which degrees of interspecific morphological similarity wax and wane during development as a result of the interplay between developmental constraints and phyletic variation.

## Introduction

The enormous disparity of adult forms in the animal kingdom is underpinned by evolutionary changes in development, and developmental divergences among disparate taxa have long been the focus of comparative embryologists [1]. Among the earliest and most influential workers investigating embryonic divergence was Karl von Baer, whose laws of animal development state that “*What is common to a larger group of animals forms earlier in the embryo than what is special.*”, and “*From the most general of the formal relationships the less general is formed and so on until the most specific finally appears.*” [2]. These laws were incorporated into an evolutionary framework by Ernst Haeckel in the form of the recapitulation model [3].

Recapitulation posits that the appearance of an embryo during the early stages of development resembles early ancestral taxa, with features specific to the species arising during later stages of development [3]. Subsequent investigations of interspecific developmental variation led to the establishment of the hourglass model that focuses on the timing of the appearance of the most conserved developmental stage [4]. The hourglass model posits that developmental variation is greater at early developmental stages, becomes reduced at a phylogenetically conserved mid-embryonic stage (known as the phylotypic stage), and increases again during later stages of development [3]. Developmental divergence, that is, the pattern of accumulated developmental differences among clades [5], has recently been argued to be recapitulatory following the phylotypic stage, in which lineage-specific features appear after features conserved across a broader phylogenetic scale [3]. While much ongoing controversy regarding the hourglass model and recapitulation has focused on the degree of conservation of the mid-embryonic phase [6–9], less attention has been paid to the later stages of development in which lineage-specific features arise [1]. Moreover, a number of examples of early developmental divergence leading to convergent later stages have been documented [10], supporting the idea that developmental divergence leading to adult variation may occur at multiple stages [5].

Examining developmental divergence between clades at late developmental stages is necessary to test these concepts, with a focus on the development of shared features at various hierarchically inter-nested phylogenetic levels, i.e. synapomorphies and autapomorphies. Such developmental comparisons will enable von Baer’s hypothesis, that developmental differences accumulate as development proceeds, to be tested, along with the prediction of the recapitulatory pattern that common, interclade features only appear at relatively early stages following the phylotypic stage, or at least before unique, clade-specific features appear. Towards exploring these predictions, in this study we carry out late-stage developmental comparisons of cranial embryonic osteogenesis in four bird species belonging to the clade Galloanserae—a clade exhibiting dramatic cranial differences among its major constituent subclades.

### Craniofacial developmental divergence in Galloanserae

Galloanserae is one of the oldest, most iconic, and most economically important clades of crown-group birds and is divided into two major subclades: landfowl (Galliformes) and waterfowl (Anseriformes) [11, 12]. These two clades are each other’s closest living relatives, yet Anseriformes exhibit numerous specialisations for aquatic habits, while Galliformes are terrestrial. These attributes have resulted in the acquisition of numerous anatomical differences between these clades, making them ideal candidates for studying the patterns of developmental change leading to their disparate adult morphologies. Moreover, some galloanserans have been extensively studied in embryological research, such as the chicken (*Gallus gallus*) (see review [13]), Japanese quail (*Coturnix japonica*) [14–16], and mallard (*Anas platyrhynchos*) [17–19], emphasising the tractability of focusing on Galloanserae as a case study.

One of the most variable anatomical regions between galliforms and anseriforms is the skull, typified by the strikingly divergent beaks of chickens and bills of ducks. Previous studies of galloanseran cranial development, specifically in ducks, quails, and chickens, have found that facial shape differences among these species become pronounced after the onset of facial prominence formation [20, 21]. Facial prominences are protuberances that are precursors to the upper and lower jaws, which include the frontonasal, maxillary, lateral nasal, and mandibular prominences [22]. After Hamburger and Hamilton (HH) stage 17, chickens and quails exhibit narrower frontonasal prominences, shallower forebrains, and larger maxillary prominences relative to ducks [21]. Furthermore, at HH stages 22–27, phenotypic trajectory analysis of craniofacial shape showed that chickens, quails, and ducks have distinct shape trajectories [23]. These divergent interclade trajectories include the formation of a mediolaterally wider frontonasal prominence in ducks and a narrower prominence with a pointed tip in chickens and quails [21]. Moreover, within Galliformes, the significantly different trajectories between chickens and quails are thought to be due to size differences [23]. Furthermore, trajectory analysis of facial shape at HH stages 22–30 has shown convergence during the fusion of the facial prominences [24]. The fusion of facial prominences constrains facial shape variation because of the likelihood of clefting if prominences fail to fuse correctly [24]. After prominence fusion, galloanseran facial shape diverges until adulthood, yielding numerous differences in cranial morphology, including a mediolaterally wide rostral extension of the upper and lower jaws in many ducks relative to the comparatively narrow, ventrally pointed beaks of most Galliformes [24, 25].

The early to mid-embryonic shape differences documented between chicken, quail, and duck account for some of the adult shape disparity within Galloanserae. However, the extent to which these embryonic differences are amplified, reduced, or maintained until hatching or adulthood has not been investigated, despite the fact that early diverging trajectories can converge at later stages [10, 26, 27]. Moreover, investigations of early embryonic differences do not account for the appearance of osteological features that are shared between galliforms and anseriforms (i.e. synapomorphies of Galloanserae), which hinders an examination of the predictions of von Baer’s laws on late-stage development.

We attempt to fill these gaps by studying skull osteogenesis to elucidate how the distinctive osteological features of the galliforms and anseriforms develop [28–33]. Different synapomorphies of Galliformes and Anseriformes have been reported by different workers. Mayr (2017) [30] listed ovate-shaped basipterygoid processes, a mandibular process of the quadrate with two ventral condyles, and elongated retroarticular processes of the mandible as galloanseran synapomorphies. Olson and Feduccia (1980) [28], in an outdated argument against galloanseran monophyly, highlighted the strikingly divergent skeletal features of anseriforms and galliforms, inadvertently providing a list of galliform and anseriform synapomorphies, respectively. Distinctive features of the galliform skull relative to that of anseriforms include unfused bones of the beak, fused postorbital and zygomatic processes, and galliform-specific shapes of the palatine, quadrate, pterygoid, auditory region, and beak [28]. As for anseriforms, they listed fused bones of the bill, a fused lacrimal and frontal, the presence of occipital fontanelles, and anseriform-specific shapes of the palatine, quadrate, pterygoid, auditory region, and bill [28]. Furthermore, Cracraft (1988) [33] proposed several cranial characters supporting the monophyly of Galloanserae, though these were disputed by Ericson (1996) [34].

Further phylogenetic analyses, especially with the inclusion of molecular data, have confirmed the monophyly of Galloanserae [35], and recent studies (e.g. [32]) continue to clarify osteological variation within the clade (Table 2). For instance, Olsen (2017) [31] emphasised that the bills of herbivorous waterfowl, such as the swan goose *Anser cygnoides*, are dorsoventrally deeper with a mediolaterally wider caudal end than filter-feeding waterfowl such as mallards. Thus, multispecies comparisons within both Galliformes and Anseriformes may further illustrate the origins of osteological variation within these groups.

Illuminating how the distinctive osteological features of the anseriform and galliform skull form requires a detailed comparative examination of galloanseran skull formation. However, previous studies of skull formation have focused on the embryonic tissue from which bones are derived [36], their modes of ossification (chicken: [37], Japanese quail: [15, 16], blue-breasted quail: [38], mallard: [39], goose: [40]), and their sequence of ossification (Galliformes: [41, 42], Anseriformes: [18]). Ossification sequence studies have found that galloanseran cranial ossification sequences are mostly congruent [18], but the shapes of these ossifications have, for the most part, only been described in chicken ([42–44]). Consequently, a descriptive comparison of skull formation among Galloanserae is needed.

A comparison of skull development in different clades requires the establishment of a skull-specific staging system to effectively describe the shapes of each ossification at relatively similar time-points during skull development in different species. A skull-specific staging system could rely on available staging systems, which use shared external morphological features to stage embryos to determine the relative timing of the onset of cranial ossification in different species. Arnaout et al. (2021) [42] established a skull staging system for chicken based on the Hamburger and Hamilton staging system [45]. However, the Hamburger and Hamilton staging system has limited applicability to quails, mallards, and geese, because the skull forms at the later stages of development and differences among species are amplified at these stages [25, 46]. Consequently, a new skull staging system is needed that is applicable to Galloanserae as a whole.

## Materials and methods

### Specimen acquisition

Seventy-seven fertilised Galloanserae eggs were obtained from a variety of sources: 26 chicken (*Gallus gallus*) eggs from MedEggs (Norwich, UK), 20 Japanese quail (*C. japonica*) from the Roslin Institute (Midlothian, UK), 21 mallard eggs and 10 swan goose (*A. cygnoides*) eggs from Anglia Waterfowl farm (Ipswich, UK). The eggs were incubated in two Brinsea incubators (Ovation 56 and 28 eco) according to the manufacturer’s guidelines, at 37.5 °C and humidity of 50% RH for Galliformes eggs and 45% RH for Anseriformes eggs. The eggs were incubated until reaching skull osteogenesis stages, which begin after 8 days of incubation in chickens [42] and quails [15], 11 days in mallards [18], and 14 days in geese (approximated from [18]). Subsequently, embryos were extracted from the eggs, following UK Home Office Regulatory guidelines, at daily or 2-day intervals depending on the species. Additionally, seven late-stage goose eggs were obtained with deceased embryos inside of them from Anglia Waterfowl farm.

### Specimen staging and imaging

Live embryos were extracted at the start of skull osteogenesis, then staged based on their species-specific staging systems (chicken: [45], quail: [46], mallards and geese: [25] and [47]). Moreover, the seven deceased goose embryos were staged and their ages approximated following [47].

After staging the embryos, they were fixed in paraformaldehyde (PFA) (Sigma-Aldrich, #441244) overnight, then dehydrated with an ethanol series (25%, 50%, 75%, 100%). In some specimens the heads were isolated post-dehydration, while others were isolated during the extraction process according to UK Home Office Regulatory guidelines. The embryonic heads were scanned using a Nikon XTEK H 225 ST µCT scanner at the Cambridge Biotomography Centre using appropriate scanning parameters maximising bone contrast (voltage: 90–170 kV, exposure time: 354–500 ms, current: 50–225 $\mu A$). Afterwards, some image stacks were denoised using the Non-Local Means Filter module in Avizo software 2019.3 with specific parameters (Spatial Standard Deviation: 5–10, intensity standard deviation 0.2–0.5, Search window [px]: 10, and Local Neighbourhood [px]: 3–5). Finally, the bones were digitally segmented using Dragonfly 2022.1.

### Skull staging system

**Fig. 1:**
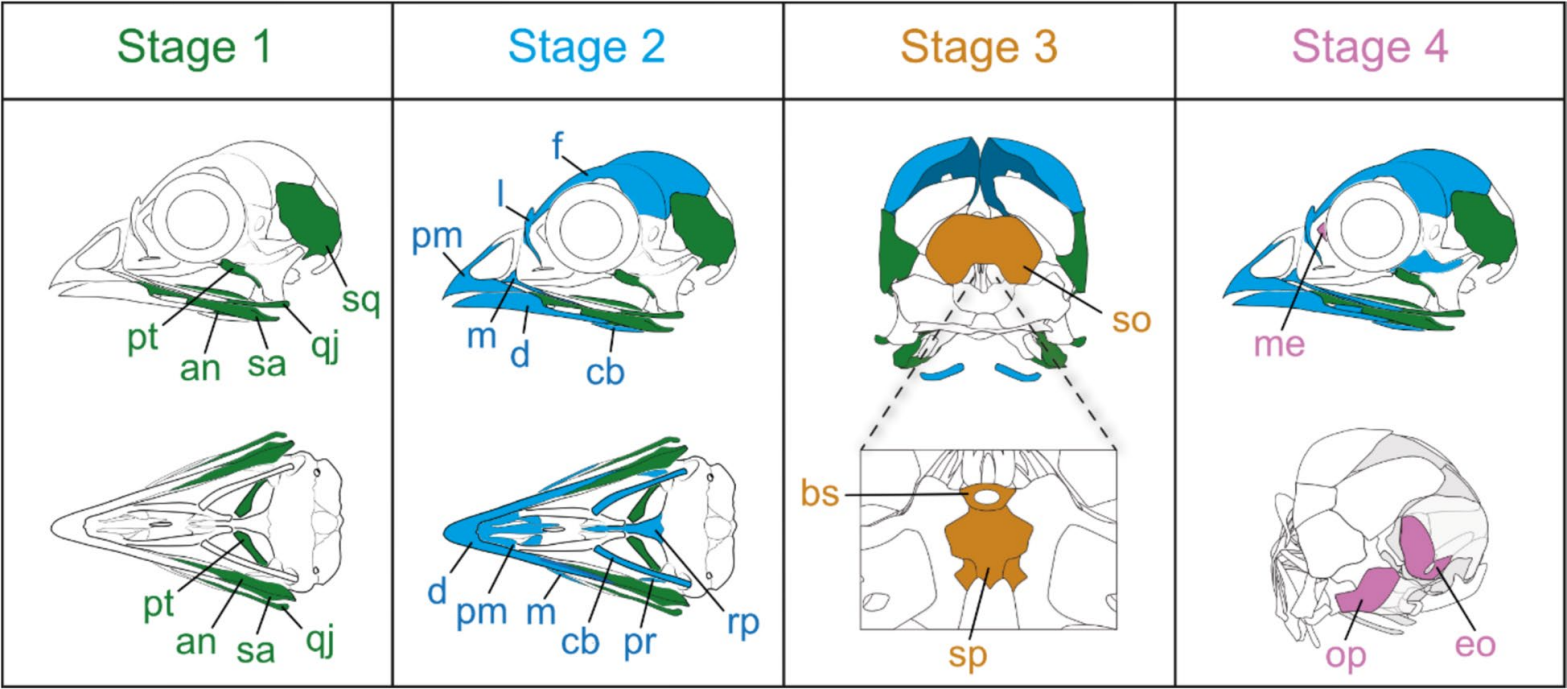
Staging system for skull osteogenesis illustrated with chicken skulls. The appearance of a coeval collection of elements within the same embryo was used as a method of discretising the continuous process of skull osteogenesis. Skulls shown at skull stages 1 and 2 are in lateral (top) and ventral views (bottom). At skull stage 3, a caudal view of the skull is illustrated on the top and the endocranium is shown in rostral view on the bottom. At skull stage 4, a lateral view is shown on the top and a caudolateral view is shown on the bottom. Abbreviation list in the main text

To generate a skull staging system we relied on the commonly used method of identifying similar features among embryos of different species and assessing the order of formation of their shared features [45] and [25]. The earliest shared features define the earliest embryonic stages, and subsequent shared features define later stages. The shared features we used to define early to late stages of skull osteogenesis were the ossification of a collection of homologous elements and their order, i.e. the ossification sequence. The process of skull osteogenesis was divided into five stages, referred to as ‘skull stage #’, based on the co-ossification of a set of homologous elements in all four species (see Fig. 1 for the full set of elements diagnosing each skull stage). Each stage is defined by the appearance of the same set of elements across our taxon sample. For example, in skull stage 1 the angular, supraangular, squamosal, quadratojugal, and pterygoid appear in all four species. However, due to variation in the rate of ossification between different species, some embryonic stage 1 skulls contained some stage 2 elements (Table 1). However, skull stage 5 was uniformly defined as the hatching stage, at which the embryos were fixed hours before hatching.

### Anatomical abbreviations used in figures

The nomenclature of the elements is based on [44]. Abbreviations are as follows: angular-an; basioccipital-bo; basiparasphenoid-bp; basisphenoid-bs; ceratobranchial-cb; dentary-d; epiotic-eo; exoccipital-ex; frontal-f; jugal-j; lacrimal-l; lagner otolith-lo; maxillary-m; mesethmoid-me; nasal-n; orbitosphenoid-ob; opisthotic-op; palatine-pl; parietal-p; prearticular-pr; premaxillary-pm; prootic-po; pterygoid-pt; quadrate-qj; quadratojugal-qj; rostroparasphenoid-rp; saccular otolith-sao; sclerotic ring-sr; sellaparasphenoid-sp; splenial-sl; squamosal-s; supraangular-sa; supraoccipital-so; utricular otolith-uo; vomer-v.

## Results

We provide comprehensive, element-by-element comparative descriptions of galloanseran skull osteogenesis in the supplementary materials. Below, we present abridged descriptions of selected elements exemplifying general patterns of galloanseran skull osteogenesis.

### Skull stage 1

**Fig. 2:**
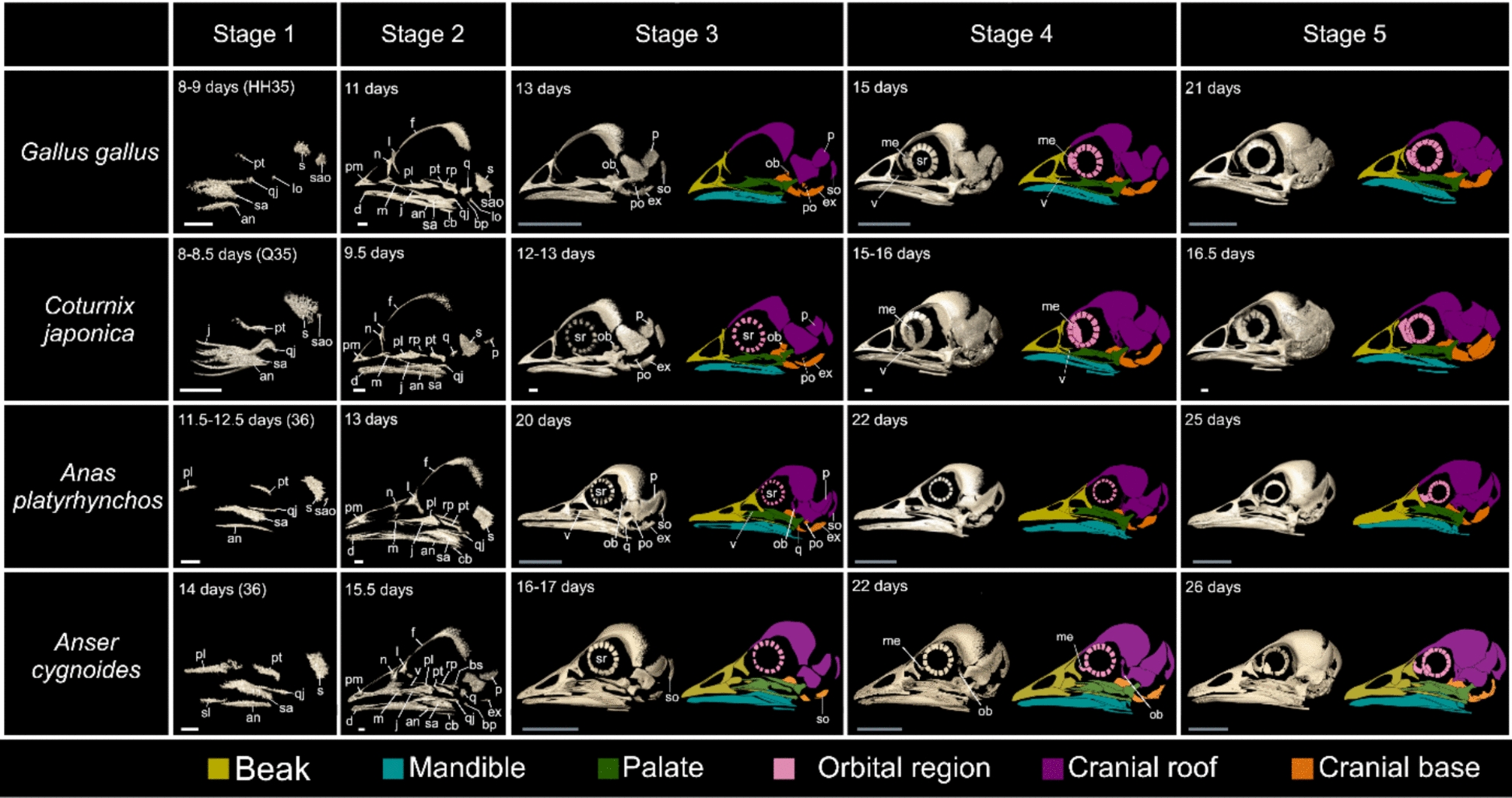
Osteogenic series of the skulls of four galloanseran species: chicken (*Gallus gallus*)*,* Japanese quail (*Coturnix japonica*)*,* mallard (*Anas platyrhynchos*)*,* and swan goose (*Anser cygnoides*). Skulls are shown in lateral view with the rostral side on the left in all panels. The orbital region refers to the mesethmoid and sclerotic ring only, and we considered the frontal as part of the cranial roof [53]. White scale bars are 1 mm, and grey scale bars are 1 cm. Abbreviation list in the main text

**Fig. 3:**
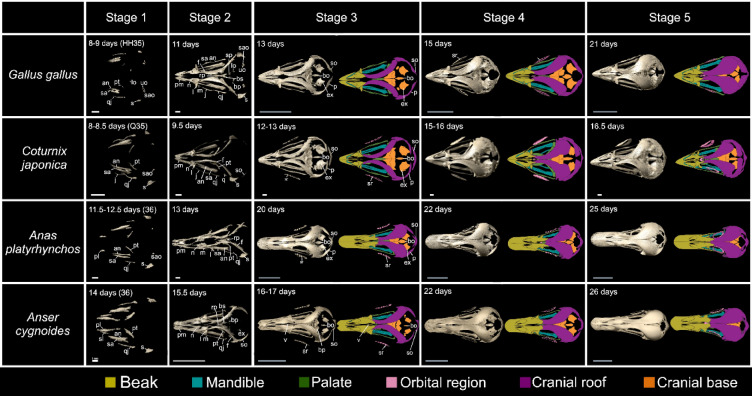
Osteogenic series of the skulls of four galloanseran species: chicken (*Gallus gallus*)*,* Japanese quail (*Coturnix japonica*)*,* mallard (*Anas platyrhynchos*)*,* and swan goose (*Anser cygnoides*). Skulls are shown in dorsal view with the rostral side on the left in all panels. The orbital region refers to the mesethmoid and sclerotic ring only, and we considered the frontal as part of the cranial roof [53]. White scale bars are 1 mm, and grey scale bars are 1 cm. Abbreviation list in the main text

**Fig. 4:**
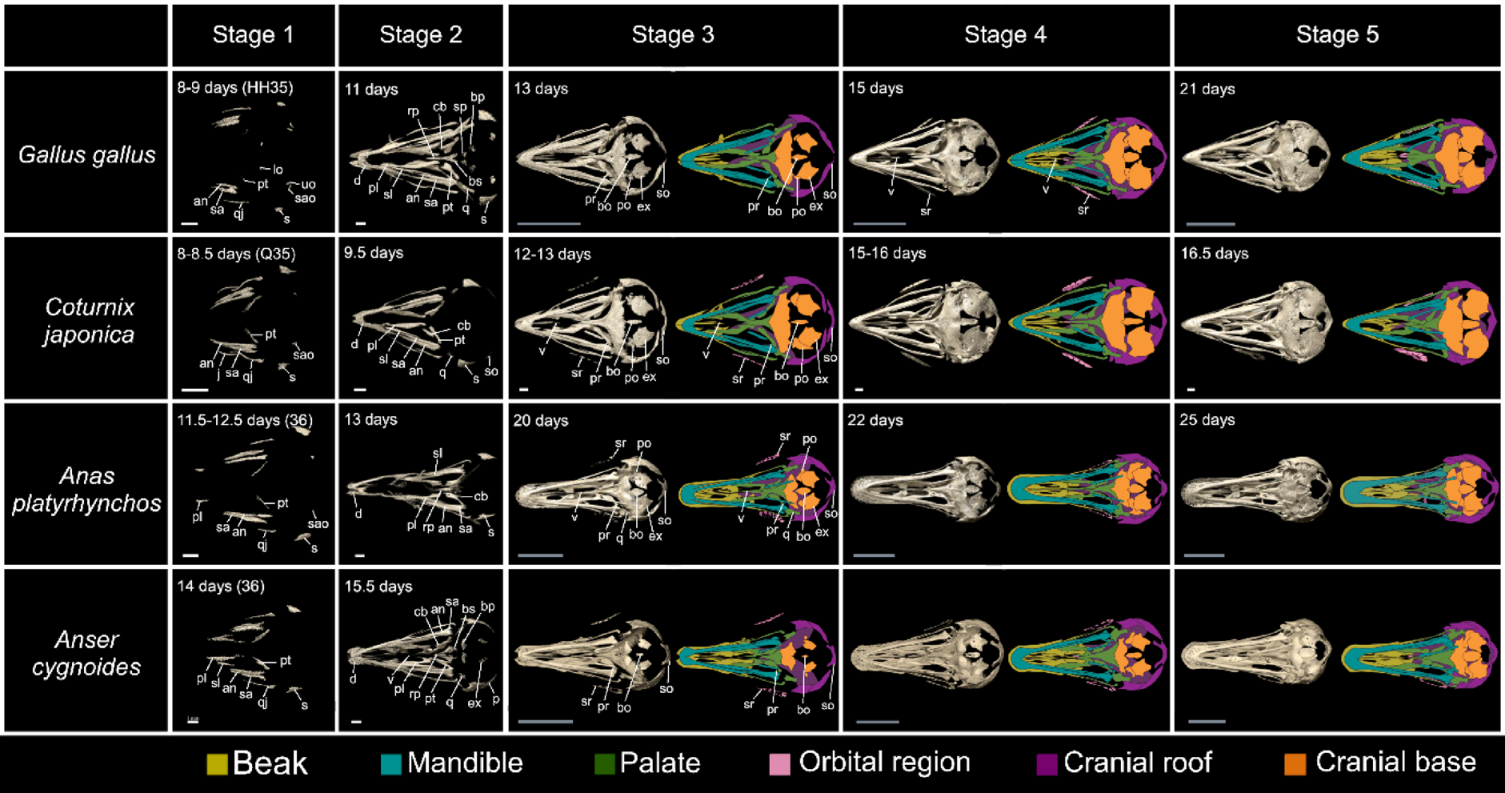
Osteogenic series of the skulls of four galloanseran species: chicken (*Gallus gallus*)*,* Japanese quail (*Coturnix japonica*)*,* mallard (*Anas platyrhynchos*)*,* and swan goose (*Anser cygnoides*). Skulls are shown in ventral view with the rostral side on the left in all panels. The orbital region refers to the mesethmoid and sclerotic ring only, and we considered the frontal as part of the cranial roof [53]. White scale bars are 1 mm, and grey scale bars are 1 cm. Abbreviation list in the main text

Skull ossification begins in chickens at HH stages 35–36 (8–10 days), quails at stages 35–36 (8–8.5 days), mallards at stages 36 (11.5–13 days), and geese at stage 36 (14 days) (Figs. 2, 3, 4 and Meshes S1–14). The elements that appear in all four species at this skull stage are the caudolateral elements, which include the angular, supraangular, squamosal, and quadratojugal, along with the pterygoid. The shapes of the angular, supraangular, and squamosal ossification centres appear to be similar across all species at this skull stage.

On the other hand, the pterygoid exhibits a combination of clade- and species-specific features. The pterygoid appears, in dorsal view, as a rostromedially and caudolaterally oriented tubular ossification (Figs. 2, 3 and Meshes S1–14). In Galliformes, the dorsal side of the pterygoid is ossified and appears, in dorsal view, as a sheet of bone with a rostral end that is mediolaterally wider than the caudal end. Moreover, in chickens, the rostral end appears, in dorsal view, to be mediolaterally wider than in quails (Meshes 3–5,7–9). However, in Anseriformes, the rostral side of the pterygoid appears, in lateral view, wedge-shaped with a mediolaterally wide rostral end, with a rostral process (sensu [28, 48]) extending from the rostromedial side. The caudal end of the pterygoid is dorsoventrally deeper and mediolaterally narrower than its rostral end, and is directed ventrolaterally (Meshes S11 & 13). Moreover, in mallards, the rostral process appears long and curves such that it parallels the medial plane of the skull, while in geese, the rostral process is shorter and points more medially. Caudally, the mallard pterygoid is narrower and more laterally curved than that of the goose when viewed dorsally (Meshes S11 & 13).

### Skull stage 2

This skull stage occurs in chickens at HH stages 37–38 (11–12 days), quails at stages 37–39 (9.5—11 days), mallards at stages 37.5–38 (13.5–19 days), and geese at stages 36–37 (14–15.5 days) (Figs. 2, 3, 4 and Meshes S14–29). The elements that appear in all replicates of all four species are the premaxillary, nasal, lacrimal, frontal, dentary, jugal, splenial, maxillary, palatine, prearticular, rostroparasphenoid, and ceratobranchial.

Most of the elements of the skull exhibit a combination of clade and species-specific features, such as the premaxillary, which appears laterally as an arrowhead-shaped ossification with three short, caudally projecting, triangular processes: the maxillary, frontal, and palatal processes (sensu [49]) (Figs. 2, 3). Galliform-specific features are a rostrocaudally shorter premaxillary, relative to that of Anseriformes, along with a fine rostral tip, a smaller triangular body of the element, a ventrolaterally directed maxillary process, and a more dorsally directed frontal process (Meshes S14–22). On the other hand, in anseriforms, the premaxillary is rostrocaudally longer with a mediolaterally wide body with a blunt rostral tip, a dorsolaterally pointing caudal tip of the maxillary process in lateral view, and a more ventrally pointing frontal process in dorsolateral view (Meshes S23–29). As for species-specific features, in mallards, the body of the element is rostrocaudally longer with a more ventrally pointing frontal process relative to those of geese when viewed dorsolaterally (Meshes S23–29). In Galliformes, the body of the premaxillary is shorter in quails than in chickens when viewed laterally (Meshes S14–22).

However, some elements exhibit clade-specific features such as the quadrate (Fig. 2, Meshes S14–22, 24–29). The otic process (adapted from proc. otic. quad sensu [50]) is the first part of the quadrate to ossify and exhibits a tubular shape. It is dorsoventrally narrower in Galliformes than in Anseriformes in lateral view (Fig. 2, Meshes S14–22, 24–29).

### Skull stage 3

**Fig. 5:**
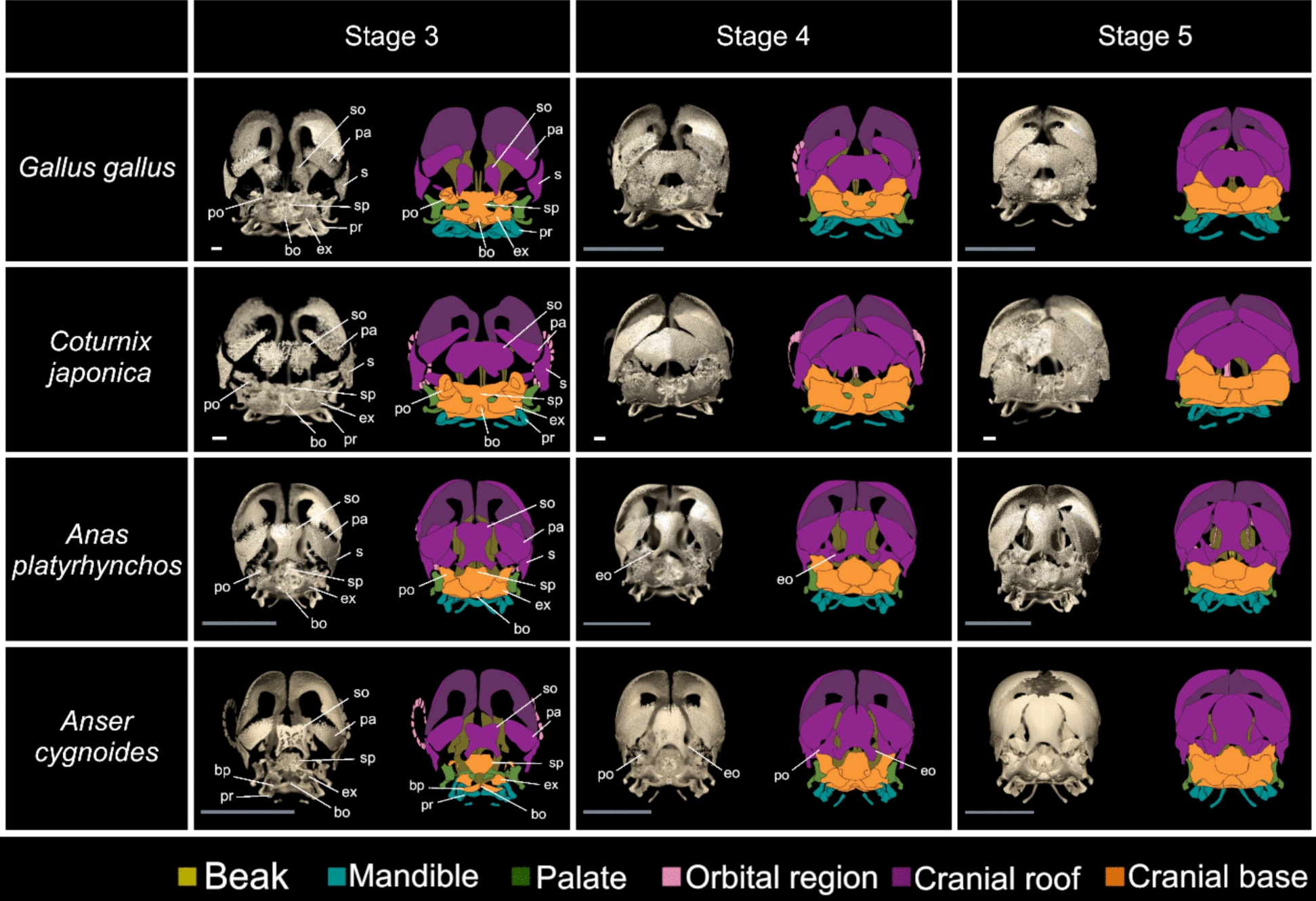
Osteogenic series of the skulls of four galloanseran species: chicken (*Gallus gallus*)*,* Japanese quail (*Coturnix japonica*)*,* mallard (*Anas platyrhynchos*)*,* and swan goose (*Anser cygnoides*). Skulls are shown in caudal view with the dorsal side on the top in all panels. The first two skull stages were omitted due to the absence of any caudal element formation. The orbital region refers to the mesethmoid and sclerotic ring only, and we considered the frontal as part of the cranial roof [53]. White scale bars are 1 mm, and grey scale bars are 1 cm. Abbreviation list in the main text

This skull stage occurs in chickens at HH stages 39–40 (13–14 days), quails at stages 40–43 (11–14 days), mallards at stages 39–41 (16–21 days), and geese at stages 38–40 (15–20 days) (Figs. 2, 3, 4, 5 and Meshes S30–52). Three new elements appear in all replicates at this skull stage: the supraoccipital, basisphenoid, and sellaparasphenoid, along with the quadrate, parietal, basiparasphenoid, alaparasphenoid, rostroparasphenoid, and exoccipital (which appear in some replicates of the previous skull stage).

Most elements of the skulls exhibited a combination of features that are shared among our entire taxon sample, along with clade- and species-specific features. For example, the maxillary process of the premaxillary appears as a triangular, caudally directed process with a fine caudal tip, in lateral view. In Galliformes, the maxillary process extends caudoventrally with a fine tip forming the caudoventral end of the beak when viewed laterally (Fig. 2, Meshes S30–40). In Anseriformes, the maxillary process extends caudodorsally towards the maxillary process of the nasal when viewed laterally (Fig. 2, Meshes S41–52). The frontal process appears mediolaterally flat with a fine caudal tip extending caudodorsally and appears proportionally longer in Anseriformes than in Galliformes when viewed dorsally (Fig. 3 and Meshes S30–52). The palatal process appears as a triangular extension on the medial side of the premaxillary extending towards the palatine and is proportionally shorter rostrocaudally in Galliformes than in Anseriformes when viewed mediolaterally (Meshes S30–52).

Regarding species-specific features, the maxillary process is dorsoventrally shallower in quails and geese than in chickens and mallards, respectively, when viewed laterally (Figs. 2 and Meshes S30–52). Furthermore, the fused rostral end of the premaxillaries is rostrocaudally shorter in geese than in mallards when viewed dorsally, and is ventrally directed when viewed laterally (Figs. 2, 3 and Meshes S41–52). In chickens, the palatal process appears mediolaterally thin in ventrolateral view, and dorsoventrally shallow and concave ventrally, but appears rostrocaudally shorter, straight, and ventrolaterally slanted in quails (Meshes S30–40). The anseriform palatal process resembles that of the chicken but is proportionally longer rostrocaudally when viewed ventrolaterally, while in mallard the process appears dorsoventrally flatter and proportionally longer rostrocaudally than in geese when viewed ventrolaterally (Meshes S41–52).

Similarly, the lacrimal forms some species-specific features at this stage, as well as a galloanseran synapomorphy in mallards and quails [32]: at its caudomedial end, a triangular, mediolaterally narrow, dorsoventrally shallow, rostrocaudally short, and caudally pointing orbital process appears in dorsolateral view (Table 2, Chr.12.1; Meshes S34–45). Regarding species-specific features, the galliform lacrimal head appears mediolaterally wider and dorsoventrally deeper, with more rounded dorsal and lateral edges in chickens than in quails, as seen in rostral view (Meshes S30–40). Within Anseriformes, the caudal end of the lacrimal head appears, in lateral view, to be of a similar flat rectangular shape in mallards and geese at this skull stage but makes up a larger proportion of the head in geese than in mallards. The rostral end of the lacrimal head appears rostrocaudally longer in mallards than in geese when viewed laterally (Fig. 2, Meshes S41–52).

However, some elements, such as the supraoccipital, exhibit clade- and species-specific features at this skull stage (Fig. 5, Meshes S30–52). In Anseriformes, it exhibits a mediolaterally wide, rounded dorsal side, which decreases in width towards its ventral side, as seen in caudal view. Laterally and ventrally, the basioccipital has concave edges with a ventrolaterally projecting process observable in caudal view (Fig. 5, Meshes S41–52). In Galliformes, the supraoccipital appears as two ossification centres, right and left; these are fused in some replicates, as seen in caudal view (Fig. 5, Meshes S30–40). The fused supraoccipital is a mediolaterally wide, trapezoidal sheet of bone with rounded corners and mediolaterally wider rostral than caudal edges, as seen in caudal view. Its ventral edge appears concave and is mediolaterally wider but of similar shape to its counterpart in Anseriformes, as seen in caudal view. At the species level, the supraoccipital appears proportionally wider mediolaterally in geese and quails than in mallards and chickens, respectively, as seen in caudal view (Fig. 5, Meshes S30–52).

Other elements continue to exhibit clade-specific features such as the quadrate. It extends in rostral and ventral directions in all four species, displaying some clade-specific features including an anseriform synapomorphy. The otic process appears dorsoventrally longer in Galliformes than in Anseriformes when viewed laterally (Fig. 2, Meshes S30–52). On its caudomedial side, a large foramen appears caudally in mallards and some goose replicates, a synapomorphy of Anseriformes [32] (Table 2, Chr.51.1; Meshes S41–46,48–50).

### Skull stage 4

This skull stage occurs in chickens at HH stages 40–44 (15–18 days), quails at stage 44 (15–16 days), mallards at stage 41–42 (22–23 days), and geese at stage 42 (26–27 days) (Figs. 2, 3, 4, 5 and Meshes S53–71). Moreover, at this skull stage, the skull approaches its shape at hatching, with a few elements appearing in all four species, including the mesethmoid, epiotic, and opisthotic. Several elements that appear in only some replicates of the previous skull stage appear in all replicates at this skull stage, including the vomer, orbitosphenoid, prootic, and articular.

Similar to the previous stage, most of the elements of the skull exhibit a combination of clade and species-specific features, such as the premaxillary. The premaxillary extends caudally in all four species examined. In mallards, the maxillary process extends further caudally than in geese, nearing the caudal edge of the maxillary process of the nasal when viewed laterally. Similarly, the palatal process extends further caudally in mallards, relative to the condition in geese, nearing the caudal maxillopalatine process of the maxillary when viewed ventrally (Meshes S64–71). In Galliformes, the palatal process appears ventrally longer and mediolaterally wider in quails than in chickens (Meshes S53–63).

Other elements exhibit clade-specific features such as the pterygoid. The pterygoid exhibits a similar shape to that of the previous skull stage (Meshes S64–71). However, in Galliformes, its rostromedial component appears wider in ventral view, forming a more defined basipterygoid contact (Meshes S53–63).

A few elements such as the lacrimal also exhibit features common among our entire taxon sample. The lacrimal exhibits a similar shape in chickens and quails at this skull stage due to the pointed dorsolateral extension of the lacrimal head, seen in rostral view (Fig. 3, Meshes S53–55,57–60,69,71). A galloanseran synapomorphy, the apomorphically short orbital process, appears in some chicken and goose replicates at the caudomedial edge of the lacrimal at this stage (Table 2, Chr.12.1; Meshes S34–45).

### Skull stage 5

The final embryonic skull stage occurs at the hatching stage. In chickens, this occurs at HH stage 45 (21 days), quails at stage 45 (16.5 days), mallards at stage 43 (26 days), and geese at stage 42 (28 days) (Figs. 2, 3, 4, 5 and Meshes S72–84). All the cranial elements have ossified by this skull stage with the notable absence of the retroarticular, postorbital, and basipterygoid processes, along with the mandibular condyles of the quadrate (Meshes S72–84). On the other hand, a galliform synapomorphy becomes clearly discernible at this stage: the presence of a rostromedial foramen on the otic process of the quadrate, which is visible in caudomedial view (Table 2, Chr.52.1; Meshes S72–77).

## Discussion

### Galloanseran skull osteogenesis

Comparing skull osteogenesis across four galloanseran species reveals at least four potentially variable aspects of cranial element formation: the location of initial ossification within each element, the direction of ossification progression, the proportions of an element relative to others, and the shape of each element. The location of the initial ossification within most elements was similar among all four species. For example, the ossification of the quadrate begins from its dorsocaudal end, i.e. the otic process, and the premaxillary ossification begins in the prenasal region in all four species (Fig. 2). This similarity in the localisation of the initial ossification appears to extend to more distantly related bird and non-avian reptile taxa for which data exist, including Budgerigar *Melopsittacus undulatus* (Psittaciformes), Java Sparrow *Lonchura oryzivora* (Passeriformes; [51]), Emu *Dromaius novaehollandiae* (Palaeognathae; [52]), American alligator *Alligator mississippiensis* (Crocodylia; [53] and [54]), and brown anole *Anolis sagrei* (Lepidosauria; [55]).

Another similarity in skull osteogenesis among the four galloanseran species studied is the direction of ossification progression within each element. For example, the quadrate, parietal, supraangular, angular, and quadratojugal ossify in a caudal to rostral direction in all four species, while the frontal and skull base ossify in a rostral to caudal direction. This similarity in the directionality of osteogenesis is also discernible in the skull development of Budgerigar, Java sparrow [55], Emu [56], American alligator [57], and brown anole [59]. Conservation of the location of ossification initiation and the direction of ossification progression within most elements implies the presence of conserved spatial signals for initiating osteogenesis across Galloanserae. These signals may emanate from surrounding tissues such as the brain [57] and chondrocranium [56].

### Variance in element disparity

The cranial osteogenesis programme produced a spectrum of shapes among homologous osteological elements across our sample, with varying degrees of disparity. Some elements, such as the frontal, basisphenoid, sellaparasphenoid, and rostroparasphenoid, were very similar among all sampled galloanserans. Two galloanseran synapomorphies appeared during the osteogenesis process: a short orbital process of the lacrimal and a premaxillary with a length < 50% of skull length [32]. These conserved features indicate that the osteogenesis programme for these elements is largely constrained within Galloanserae. However, most other cranial elements were more disparate, particularly in comparisons between Galliformes and Anseriformes rather than within these clades. These disparate morphologies were apparent throughout the process of skull osteogenesis, from the initial shape of ossifications to the shape of these elements at more mature skull stages, with the relative proportions of these elements also exhibiting pronounced variability among the taxa investigated. For example, the shape of the body of the premaxillary appeared long and flat in mallards and geese but was short, triangular, and rostrally pointed in chickens and quails (Figs. 2, 3). These observed disparities in element geometry between Anseriformes and Galliformes extend to other galloanserans (muscovy duck *Cairina moschata*) for which data on skull osteogenesis exist [55].

**Fig. 6:**
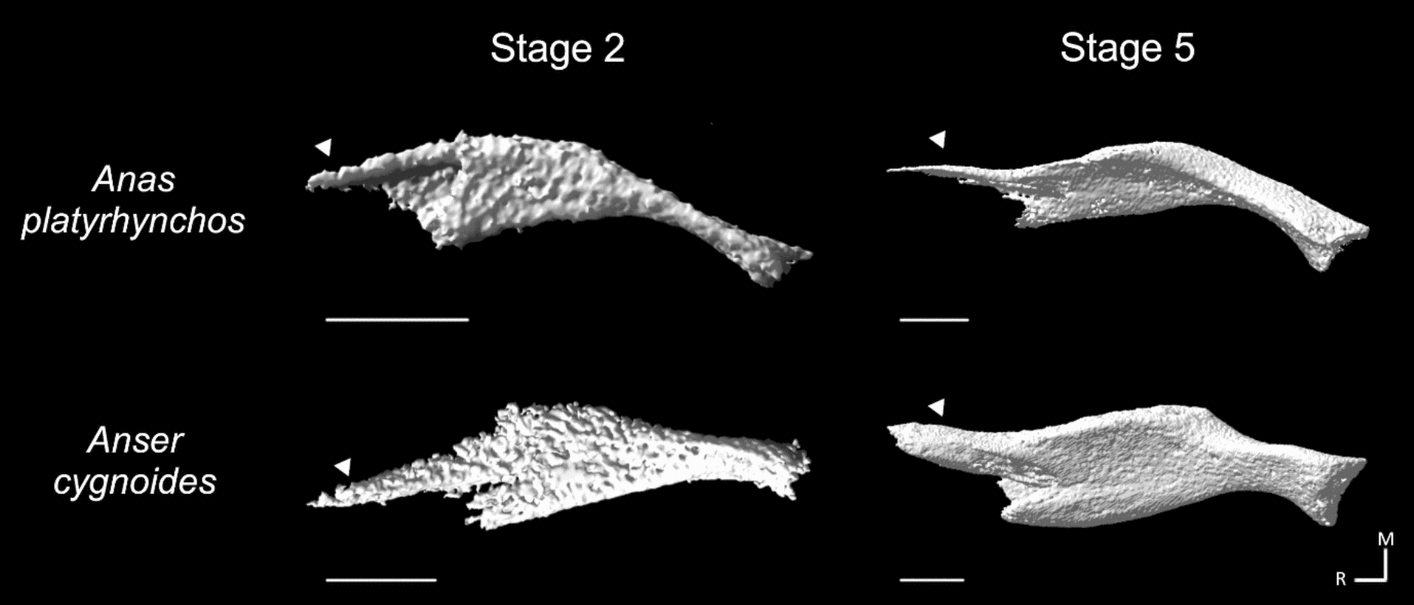
Developmental system drift in the formation of the rostral process of the pterygoid in mallard (*Anas platyrhynchos*) and swan goose *(Anser cygnoides*). The rostral process (arrowhead) forms lateromedially in geese, while in mallards it forms mediolaterally and remains on the medial side of the pterygoid. Scale bar is 1 mm; *R* rostral, *M* medial

Constraints on the variation in skull osteogenesis among these galloanserans are evident by the appearance of some anseriform and galliform synapomorphies (Skull stages 3–5, Table 2 Chr. 51.1 & 52.1), and shape convergence in pterygoid formation in goose and mallard. The rostral process of the pterygoid forms in a lateral-to-medial direction in geese, but in a medial-to-lateral direction in mallards (Fig. 6). At the initial point of ossification, the rostral process is more disparate in form (Fig. 6, Skull stage 2) than at hatching (Fig. 6, Skull stage 5). This convergence in shape appears to represent an example of developmental system drift in which different developmental pathways or types of tissue in different species can lead to the formation of similar structures [57, 58].

Other cranial features appeared to be relatively less constrained, instead exhibited species-specific patterns, such as the orientation of the frontal process of the premaxillary in geese and the elongate rostral process of the pterygoid in mallards.

### Relative timing of osteological feature formation

Besides variance in the extent of constraint on element formation, the relative order of appearance of unique species-specific and shared galloanseran features was heterogeneous. Some elements, such as the premaxillary and maxillary exhibited a combination of clade- and species-specific features in the early stages of ossification, while in the later stages only species-specific differences were observed. Other elements, such as the squamosal, exhibited both clade- and species-specific differences at early and later stages. Notably, the short orbital process of the lacrimal, which is shared among all Galloanserae, appeared after the formation of both clade- and species-specific features of the lacrimal.

Our results illustrate pronounced heterogeneity in the order of formation of common, highly constrained features, as well as unique, less constrained features. This heterogeneity raises questions about the factors that dictate the order of appearance of these osteological features. We suggest that the order in which these osteological features appear may be largely driven by two factors: the ossification sequence of a skull and the direction of ossification within skull elements, rather than the phylogenetic breadth of these features as implied by von Baer’s laws. For example, the influence of ossification sequence is illustrated by the ossification of the premaxillary in our taxon sample (an element bearing pronounced species-specific features), which occurs at skull stage 2, before the appearance of the short orbital process of the lacrimal at skull stage 4 (which is an invariant feature across our taxon sample). The influence of the direction of ossification within skull elements is exemplified by the ossification of the lacrimal, which seems to progress rostrocaudally with the appearance of the clade-specific lacrimal head at skull stage 2, preceding the appearance of the more caudally positioned short orbital process.

### Element homology

Our comparison of skull osteogenesis across Galloanserae raises questions regarding the homology of certain elements within the clade. The articular bone in chicken skulls ossifies without a suture separating it from the prearticular, as has been documented previously [44], which may suggest that the articular–prearticular complex in chicken should be regarded as a single fused element, following the logic of some recent authors (e.g. [59]). Another example is the formation of the occipital foramen in Galliformes, which resembles the anseriform occipital fontanelle. In chickens and quails, the region medial to the anterior semicircular canal appears narrower than the anseriform occipital fontanelle in rostral view, but exhibits a similar shape, suggesting that this fontanelle may not genuinely be absent in Galliformes (*contra* [28]).

### Galloanseran craniofacial divergence

Observed osteological disparity between Galliformes and Anseriformes at the pre-hatching skull stages is reflective of developmental divergences arising at earlier stages. The interclade disparity in the shape of the frontonasal prominence that appears at HH stage 27 [23] likely corresponds to the disparity in the shape of the premaxillary and nasal appearing at skull stage 2. Moreover, the interclade difference in forebrain depth [23] corresponds to the variance in the orientation of the postorbital domain of the frontal appearing at skull stage 2, which wraps around the forebrain [53]. As for mandibular disparity, mallards exhibit a greater number of mandibular neural crest cells and a faster rate of proliferation [60], as well as reduced bone absorption relative to quails [61], corresponding to differences in the relative length of the mandibular elements appearing at skull stage 2. However, the exact correspondence between early developmental disparity and embryonic osteological disparity needs further analysis and quantification. Moreover, the role of chondrocranium disparity in reflecting early morphogenetic differences and its impact on osteological disparity also require further examination [62–64].

In addition to reflecting early craniofacial morphogenetic disparity, skull formation sets the stage for the post-hatching developmental variation that leads to the adult cranial disparity observed in Galloanserae. The beak shape disparity appearing at skull stages 2 and 3 corresponds to the adult disparity typical of Anseriformes and Galliformes (Fig. 2). Moreover, the characteristically deep bill of adult geese [31] also appears at skull stages 2 and 3 (Fig. 2). Caudal to the beak, certain divergent features of the adult lacrimals of galliforms and anseriforms correspond to the disparity seen during skull osteogenesis (Fig. 2). The galliform lacrimal has a wide head and a narrow, pointed foot, features that are displayed at skull stages 3 and 4 [11] (Fig. 2). Similarly, the adult anseriform lacrimal has a wide head and narrow descending process [11]; both features appear at skull stages 3 and 4 (Fig. 2). Caudal to the lacrimal, some of the divergent features of the adult braincase of Galliformes and Anseriformes appear at skull stages 3–5 (Figs. 2, 3, 4, 5). The shallow braincase of Galliformes, with its wide supraoccipital, appears at skull stages 3–4, which contrasts with the narrow and deep braincase of Anseriformes with large fontanelles that also appear at skull stage 3–4 [28] (Figs. 2, 3, 4, 5). Caudal to the braincase, some of the disparity in the adult mandible appears at skull stages 2–3 (Fig. 4), which include the proportionally shorter dentary in quails relative to those of the other species examined, the rostrally pointed tip of the galliform dentary, and the flat tip of the anseriform dentary.

Despite the role of skull osteogenesis in establishing patterns of adult cranial disparity, numerous synapomorphies did not appear during the skull formation stages (Table 2). Some of these synapomorphies might originate at earlier stages, such as characters of the quadrate and retroarticular process (Table 2), while others might arise as a result of post-hatching bone modifications that form in response to the function of the skull, such as the basilar tubercles (Table 2, Chr. 25.1). A full determination of the origination of each synapomorphy would appear to demand a complete examination of both pre-osteogenesis and post-osteogenesis phases of head formation from early neurulation through to the adult stage.

### The ‘braiding hourglass’ model

**Fig. 7:**
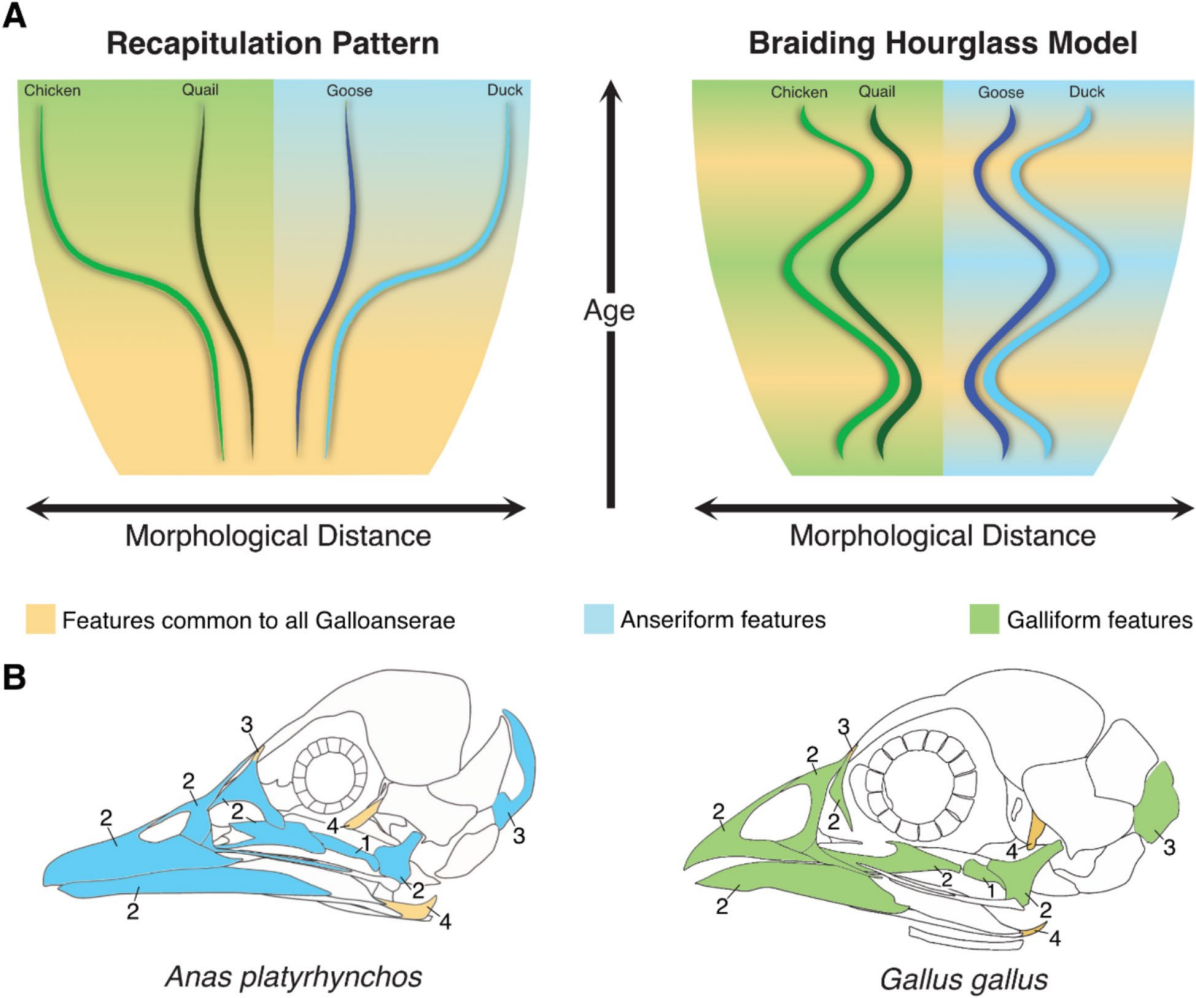
**A** Alternative models of developmental divergence between Galloanserae subclades. The recapitulatory pattern (left) predicts that features common to all members of Galloanserae (yellow) should appear before features specific to the subclades Galliformes (green background) or Anseriformes (blue background). Likewise, developmental differences between species should accumulate as development proceeds. By contrast, the braiding pattern states that features common to all members of Galloanserae can form at the same time, or even after, species-specific features; and therefore, that developmental similarities between clades may wax and wane as development proceeds. **B** Illustrations of skulls, in lateral view, of mallard (*Anas platyrhynchos*) and chicken (*Gallus gallus*) at post-hatching stage. The numbers shown represent the order of ossification of elements (or regions within an element)

The appearance of clade-specific features at a variety of stages of skull osteogenesis, along with the occurrence of developmental system drift, casts doubt on the primacy of von Baer’s Laws and the recapitulation concept in the context of galloanseran craniofacial divergence. Some of von Baer’s laws imply that galloanseran synapomorphies should appear in the embryo before subclade- and species-specific features. Similarly, the recapitulation pattern suggests that the accrual of developmental divergences between clades should resemble evolutionary transitions through their phylogenetic history, and therefore that distinctive clade-wide features should appear before species-specific differences (Fig. 7B). Given that skull osteogenesis occurs during the later stages of embryonic development, it would be expected that cranial galloanseran osteological synapomorphies would appear before galliform or anseriform synapomorphies, followed by species-specific features. Previous work on limb development in passerine birds and avian nasal capsule formation has also failed to support von Baer’s laws [65].

Most galloanseran, galliform, and anseriform cranial osteological synapomorphies did not form during embryonic skull osteogenesis, and likely form at post-hatching stages (Table 2). Moreover, the relative timing of osteological feature formation seemed to depend on the ossification sequence or its direction of ossification as opposed to correlating strictly with a feature’s degree of phylogenetic specificity (see section above on galloanseran skull osteogenesis).

An alternative model of developmental divergence can be deduced based on several observations. The similarity of facial shape at HH stage 17 [20] is followed by phenotypic trajectory divergence [21], then convergence around HH stage 25 [24]. Subsequently, skull osteogenesis gives rise to a variety of features that vary in their degree of phylogenetic specificity. These observations imply the presence of a developmental process that diverges and converges among species at a variety of developmental stages, giving rise to a ‘braiding’ pattern (Fig. 7A). The convergence of developmental trajectories can be a product of the appearance of a similar feature in different taxa under consideration, such as the rostral process of the anseriform pterygoid which unites all crown anseriforms, while divergence is a product of the appearance of differing features in the taxa under consideration, such as the narrower maxillary process of the nasal in mallards relative to that of the swan goose.

The braiding model highlights the recurring appearance of constrained features throughout developmental sequences and suggests that developmental divergences among clades arise at a multitude of stages [5]. Some early-stage developmental distinctions, such as differences in the frontonasal prominence across Galloanserae, persist into the adult stage, indicative of high developmental penetrance [5]. By contrast, other differences, such as deviations in the formation of the rostral process of the anseriform pterygoid, ultimately give rise to similar morphologies, indicating low developmental penetrance [5]. Furthermore, the constraints that affect interclade developmental divergence seem to be phylogenetically clustered. For example, the short, deep beak of Galliformes seems to be relatively constrained within the clade, while the presence of hooked retroarticular processes of the mandible is constrained across crown group Galloanserae [32].

The braiding model envisions the developmental sequence of an organism as navigating phylogenetically clustered constraints spread throughout ontogeny, some of which are more widespread than others, producing a braiding pattern within the developmental hourglass (Fig. 7A). Moreover, interclade evolutionary developmental divergences arise from shifts from ancestral developmental sequences through the acquisition of novel developmental features of variable penetrance. These novel developmental sequences may, in turn, be accompanied by their own set of constraints. Such shifts in developmental sequences may be most clearly discernible through comparisons among fairly closely related taxa, which may illustrate the developmental changes underlying discrete anatomical shifts without the risk of these being overprinted by a greater number of changes in comparisons among more distantly related taxa.

The braiding model differs from most proposed models of developmental divergence. One of the oldest models, the funnel model, is based on von Baer’s laws and argues that the earliest stages are the most conserved stages among species, followed by a linear increase in differences between clades with age [2, 66]. The funnel model differs from the braiding hourglass model by arguing that only one stage, the earliest stage, is the conserved stage, while the braiding hourglass model accommodates multiple more conserved stages in interspecies developmental divergence. Another popular model of developmental divergence is the hourglass model, which argues for conservation of the phylotypic stage only, and increased divergence following the phylotypic stage [67]. As the braiding model posits that a variety of developmental stages can be conserved, it extends the hourglass model, though it also argues that developmental divergence may decrease between a pair of species at late stages of development. However, a more recently proposed model is the adaptive divergence model [5] that attempts to account for adaptive differences between clades. This model argues that differences at the earliest and latest stages have little impact on adult variation that may undergo selection, while small differences at the phylotypic stage can have a major impact on adult variation [5]. The braiding hourglass model has a larger scope than the adaptive divergence model, as the former seeks to accommodate both adaptive and non-adaptive features and take into account developmental differences regardless of their impact on adult variation.

Other models resembling the braiding hourglass model have been proposed for metamorphic animals (the developmental goblet model [68]), and rodent molar evolution (the inverted hourglass model [69]). Moreover, developmental convergence between closely related species, at stages later than the phylotypic stage, has been documented in cichlid chondrocranial formation [70]. These studies indicate the potential ubiquity of braiding-like patterns in the developmental divergence between clades.

### Future directions

Our *µ*CT-based approach to visualising skull osteogenesis can be compared with other methods such as alizarin red staining (e.g. [42, 71]). Alizarin red staining provides the opportunity to visualise small ossifications with a high level of accuracy that may not be as easily detected in *µ*CT scans. On the other hand, CT scanning allows for visualisation of each element without damaging the tissue, and even subsequent quantification of the shapes of ossifications. The influence of alternative methods of visualising these early ossifications on our understanding of skull osteogenesis deserves further examination.

Our results highlight additional areas in which our understanding of avian skull osteogenesis remains incomplete. For instance, the task of determining the sources of ossification signaling for each element of the skull and their degree of conservation across Galloanserae remains incomplete. Moreover, identifying the molecular and cellular programmes driving variation in ossification patterns of homologous elements in different taxa is also needed. Finally, a quantitative investigation of the degree of morphological similarity in craniofacial development from the phylotypic stage to adulthood across Galloanserae would shed additional light on the nature of the braiding pattern of development across the clade. The present work’s focus on skull osteogenesis means that relevant observations from earlier and later phases of embryonic and post-hatching development are needed for a comprehensive understanding of the developmental underpinnings of cranial disparity across Galloanserae.

### Supplementary results

#### Skull stage 1

The pterygoid also appeared in all specimens investigated except for one chicken replicate. Moreover, several additional elements appear in some replicates of each species. The palatine and maxillary appear in some replicates of all four species. The nasal appears in some chicken and quail replicates, while the splenial appears in some mallard and goose replicates. The jugal appears in some chicken replicates.

The angular, supraangular, and squamosal ossification centres appear to be of similar shapes across all species at this skull stage. The angular appears, in lateral view, as a mediolaterally narrow sheet of bone that forms on the caudolateral side of the ventral aspect of the skull (Figs. 2, 4 and Meshes S1–14). Dorsal to the angular, the supraangular appears, in lateral view, as a vertical sheet of bone with a dorsoventrally deep body and gradually tapering rostral and caudal ends (Fig. 2 and Meshes S1–14). Dorsal to the supraangular, the squamosal appears, in lateral view, as a rectangular-shaped sheet of bone with rounded corners (Fig. 2 and Meshes S1–14).

Rostral to the squamosal, the quadratojugal appears, in lateral view and in all four species, as a straight dorsoventrally narrow ossification with a fine rostral tip and a mediolaterally widened, bulbous caudal end. However, in quails, the caudal end extends ventrally, creating a concave caudal tip when viewed laterally. On the other hand, the caudal tip of the quadratojugal in all the other species extends medially when viewed laterally (Fig. 2 and Meshes S1–14). Medial to the quadratojugal, the pterygoid appears, in dorsal view, as a rostromedially and caudolaterally oriented tubular ossification with clade- and species-specific features (Figs. 2, 3 and Meshes S1–14). In Galliformes, the dorsal side of the pterygoid is ossified and appears, in dorsal view, as a sheet of bone with a rostral end that is mediolaterally wider than the caudal end. Moreover, in chickens, the rostral end appears, in dorsal view, to be mediolaterally wider than in quails (Meshes 3–5,7–9). However, in Anseriformes, the rostral side of the pterygoid appears, in lateral view, wedge-shaped with a mediolaterally wide rostral end, with a rostral process (sensu [28, 48]) extending from the rostromedial side. The caudal end of the pterygoid is dorsoventrally deeper and mediolaterally narrower than its rostral end and is directed ventrolaterally (Meshes S11 & 13). Moreover, in mallards, the rostral process appears long and curves such that it parallels the medial plane of the skull, while in geese, the rostral process is shorter and points more medially. Caudally, the mallard pterygoid is narrower and more laterally curved than that of the goose when viewed dorsally (Meshes S11 & 13).

Rostral to the pterygoid, the rostral process of the palatine appears, in dorsal view, as a long, mediolaterally wide ossification with tapering rostral and caudal ends in all four species, but also shows clade-specific features (Meshes S3, 8, 9, 11, 13). In Galliformes, the rostral process (adapted from proc. rost. pal. sensu [72]) is dorsoventrally deeper with an oval dorsal profile and tapering caudal and rostral ends. On the other hand, in Anseriformes, the rostral process has a triangular dorsal profile, when viewed dorsally, with a blunt rostral end, with the choanal lamella appearing at the caudal end. The choanal lamella appears further developed in mallards than in geese, with a dorsoventrally deep, triangular lateral side that curves caudomedially (Meshes 11&13).

#### Skull stage 2

This skull stage occurs in chickens at HH stages 37–38 (11–12 days), quails at stages 37–39 (9.5—11 days), mallards at stages 37.5–38 (13.5–19 days), and geese at stages 36–37 (14–15.5 days) (Figs. 2, 3, 4 and Meshes S14–29). The overall skull shape becomes apparent at this skull stage, with many elements appearing in all four species. Galliform skulls take on their characteristic appearance with a rostrocaudally short, dorsoventrally deep, rostrally pointed beak, a large orbit, and a mediolaterally wide and dorsoventrally shallow braincase. On the other hand, anseriform skulls exhibit a rostrocaudally long, dorsoventrally flat bill with a rounded and mediolaterally broad rostral tip, a smaller orbit, and a dorsoventrally deep and mediolaterally narrow braincase. Moreover, some species differences become apparent at this stage, such as the dorsoventrally deeper bill of geese relative to that of mallards (Fig. 2 and Meshes 23–29).

The elements that appear in all replicates of all four species are the premaxillary, nasal, lacrimal, frontal, dentary, jugal, splenial, maxillary, palatine, prearticular, rostroparasphenoid, and ceratobranchial. Moreover, several other elements appear in some replicates of each species. The quadrate, parietal, and basiparasphenoid appear in some replicates of all four species. The alaparasphenoid and exoccipital appear in some replicates of all species except for mallards. The basioccipital appears in some goose replicates.

Caudal to the premaxillary, the nasal forms as a flat sheet of bone curving laterally. In dorsal view, the body of the nasal is semi-oval shaped when viewed dorsally. Little resemblance was observed between Galliformes and Anseriformes along with some species-specific differences (Figs. 2, 3). Galliform-specific features include the appearance of a maxillary process with a thin caudal tip, a rostrocaudally short premaxillary process, and a rostrocaudally short, mediolaterally wide triangular frontal process (Figs. 2, 3 and Meshes S14-22). On the other hand, in Anseriformes, the maxillary process has a rostrocaudally wide, rounded ventral tip (Fig. 2 and Meshes S23–29), a rostrocaudally longer premaxillary process extending towards the rostral edge of the naris, and a rostrocaudally longer frontal process (Figs. 2, 3 and Meshes S23–29). Furthermore, in chickens, the body of the nasal is rostrocaudally longer, and the maxillary process is rostrocaudally wider than in quails. Most goose replicates have a premaxillary process that is rostrocaudally longer than in mallards (Meshes S23–29).

Caudal to the lacrimal, the frontal forms as a supra- and postorbital ossification with little discernible difference between anseriforms and galliforms. In Anseriformes, the postorbital end is curved more ventrally in lateral view and more medially in dorsal view (Figs. 2, 3, Meshes S14–29). Caudal to the frontal, the parietal appears, in some replicates, as a crescent-shaped, vertical ossification with a rostrolateral to ventromedial orientation, with no discernible shape differences among the four species examined (Meshes S15–22, S24–29).

Medial to the squamosal, the basiparasphenoid appears in some replicates of all species, with a rectangular or trapezoidal shape in both anseriforms and galliforms, when viewed ventrally (Meshes S14,16–18,21,22,24–26,28,29). Rostral to the quadrate, the quadratojugal exhibits some clade- and species-specific differences (Fig. 2, Meshes S14–29). In lateral view, the anseriform quadratojugal is rostrocaudally longer with a mediolaterally narrow and dorsoventrally deep body, while in Galliformes the quadratojugal is rostrocaudally shorter with a rounded cross-sectional profile. Moreover, the quadrate articulation has a rounded shape and extends medially in Anseriformes, when viewed caudolaterally (Meshes S23–29). In Galliformes, the quadrate articulation is tube-like and extends ventromedially when viewed caudolaterally (Meshes S14–22). The quadratojugal is proportionally shortest rostrocaudally in quails and longest in mallards, with intermediate lengths in chickens and geese, in lateral view (Meshes S14–29).

Rostral to the quadratojugal, the jugal in lateral view is a mediolaterally narrow splinter-like ossification with clade-specific features. In Anseriformes, the jugal is mediolaterally narrower and dorsoventrally deeper than in Galliformes, with a mediolateral thickening in the middle of its body when viewed laterally (Fig. 2 and Meshes S14–29). Moreover, the jugal appears dorsal to the maxillary-quadratojugal boundary in Anseriformes but appears lateral to the boundary in Galliformes when viewed dorsolaterally (Fig. 3, Meshes S14–29).

Medial to the jugal, the palatine exhibits a mediolaterally narrow rostral process and a choanal lamella with a concave, dorsoventrally deep rostral side in dorsolateral view (Figs. 2, 3, Meshes S14–29). It exhibits a rostrally projecting vomeral process in some replicates in dorsal view (Meshes S14,15,21,22,24–26, 28,29). The palatine exhibits clade- and species-specific features at this skull stage. In Anseriformes, the rostral process (adapted from proc. rost. pal. sensu [72]) of the palatine appears as a dorsoventrally thin, mediolaterally wide sheet of bone with a tapering rostral tip, resembling its galliform counterpart, when viewed dorsally. The pterygoid process appears at the caudomedial end of the palatine as an elongate concavity facing dorsomedially and extending caudal to the caudolateral extension of the palatine, when viewed laterally (Meshes 23–29). In Galliformes, the rostral process is mediolaterally narrow with a triangular cross-sectional profile, and the vomeral process is shorter than that in some anseriform replicates, when viewed dorsally. Moreover, in galliforms, the lateral margin of the palatine curves medially towards the pterygoid process and does not form a caudolateral extension, as seen in lateral view (Meshes S14–22). The pterygoid process appears as a dorsoventrally thick horizontal plate of bone oriented caudolaterally, with rounded caudal edges. It appears narrower mediolaterally than that of Anseriformes, when viewed dorsolaterally. Regarding species-specific features, the choanal lamella appears proportionally longer rostrocaudally in chickens than in quails in lateral view, and the pterygoid process appears longer rostrocaudally and wider mediolaterally in mallards than in geese when seen in dorsal view (Meshes S14–29).

Medial to the dentary, the splenial forms as a medially concave sheet of bone with some clade-specific features (Fig. 4, Meshes S14–29). In Anseriformes, the splenial has a dorsoventrally deep caudal side that sharply tapers towards its caudal tip when viewed medially, but tapers more gradually towards its rostral tip. Conversely, in Galliformes, the splenial has a dorsoventrally deep rostral and shallow caudal end when viewed laterally. Caudal to the splenial, the prearticular exhibits clade-specific features (Fig. 4), Meshes S14–29). In Anseriformes, the prearticular is a rostrocaudally long and dorsoventrally deep element throughout most of its length, with dorsoventrally short rostral and deep caudal ends when viewed medially (Meshes S23–29). In Galliformes, however, the prearticular is a rostrocaudally short, dorsoventrally shallow, mediolaterally thin ossification that curves medially when viewed rostromedially (Meshes S14–22). Furthermore, in chickens, the caudal end of the prearticular curves dorsomedially forming the medial side of the articular when viewed rostromedially (Meshes S14–18). In quails, by contrast, the caudal extension curves medially when observed in rostromedial view (Meshes S19–22).

Lateral to the prearticular, the supraangular exhibits clade-specific features ((Fig. 4, Meshes S14–29). In Anseriformes, it extends caudoventrally, forming a mediolaterally narrow caudal tip that terminates before reaching the caudoventral tip of the mandible when viewed caudolaterally. In Galliformes, by contrast, the supraangular is dorsoventrally deeper and extends to the caudoventral tip of the mandible when viewed caudolaterally (Fig. 4, Meshes S14–29). Ventral to the supraangular, the angular exhibits some clade-specific features (Fig. 4, Meshes S14–29). In Galliformes, the angular extends caudally, forming a mediolaterally flat caudal end with a straight lateral edge and a curved medial edge, terminating in a sharp tip in ventral view. The caudal tip of the angular joins the tip of the supraangular to form the caudoventral tip of the mandible in Galliformes when viewed ventrally. Anseriformes, by contrast, exhibit an angular with a mediolaterally wide caudal end, with a straight medial and a curved lateral edge when viewed ventrally (Fig. 4, Meshes S14–29).

Ventral to the mandibular elements, the ceratobranchial forms in all four species with slightly differing orientations between galliforms and anseriforms (Fig. 4, Meshes S14–29). In Galliformes, the element is more medially directed than in Anseriformes when viewed ventrally.

### Skull stage 3

This skull stage occurs in chickens at HH stages 39–40 (13–14 days), quails at stages 40–43 (11–14 days), mallards at stages 39–41 (16–21 days), and geese at stages 38–40 (15–20 days) (Figs. 2, 3, 4, 5 and Meshes S30–52). Three new elements appear in all replicates at this skull stage: the supraoccipital, basisphenoid, and sellaparasphenoid, along with the quadrate, parietal, basiparasphenoid, alaparasphenoid, rostroparasphenoid, and exoccipital (which appear in some replicates of the previous skull stage). Moreover, the basioccipital and sclerotic ring appear in some replicates of all species. However, several elements appear in some replicates of only some species, such as the vomer, which appears at this stage in chicken, mallard, and quail replicates. The epiotic appears at this stage in some mallard and quail replicates. The orbitosphenoid appears at this stage in some mallard and quail replicates, while the prootic appears at this stage in some quail replicates. The articular appears in some mallard replicates.

Caudal to the premaxillary, the nasal displays some species-specific differences in both anseriforms and galliforms. The frontal process appears proportionally longer rostrocaudally with a mediolaterally wider caudal tip in chickens than in most of the quail replicates when viewed dorsally (Figs. 3, Meshes S30–40, 46–52). However, the maxillary process extends further rostroventrally in chickens than in quails when viewed laterally (Figs. 2 and Meshes S30–52). In Anseriformes, the frontal process is rostrocaudally longer in mallards than in geese, and the maxillary process has a rostrocaudally longer, triangular-shaped caudal end in mallards relative to the narrower rectangular-shaped end in geese, seen in lateral view (Figs. 2 and Meshes S41–52).

Caudal to the lacrimal, the frontal ossifies in a lateral-to-medial direction when viewed dorsally, with a tapering rostrolateral tip and a mediolaterally wide, rounded caudal end in all four species (Fig. 2, Meshes S30–52). A dorsally concave sheet-like ossification with narrow rostral and wide caudal ends appears on the ventral side of the postorbital domain, visible in lateral view, and exhibits some clade- and species-specific features. In Galliformes, the ventral ossification appears rostrocaudally longer and mediolaterally wider at its caudal end than in Anseriformes in laterorostral view (Meshes S30–40). In Anseriformes, the ventral ossification is mediolaterally wider at its caudal end in mallards than in geese, with an abruptly narrowing rostral end, as seen in rostrolateral view (Meshes S41–52).

Caudal to the frontal, the parietal appears as a sheet of bone with only slightly different shapes, but very different orientations between galliforms and anseriforms. In Galliformes, the parietal is rectangular with a more caudal orientation than in Anseriformes and extends dorsal to the supraoccipital, as seen in a dorsocaudal view. On the other hand, in Anseriformes, the parietal is trapezoidal, with a dorsoventrally narrow caudal end, and extends dorsolaterally to the supraoccipital, as seen in a caudal view (Fig. 5, Meshes S30–52). Moreover, the ventral edge of the parietal appears wedge-shaped when viewed ventrally, with varying thicknesses among replicates of all four species (Meshes S30–52).

The pattern of ossification of the four components of the cranial roof (frontal, parietal, supraoccipital and squamosal) reveals clade- and species-specific features of the braincase. The galliform braincase is proportionally wider mediolaterally and shallower dorsoventrally than in Anseriformes, and the quail braincase is mediolaterally wider and dorsoventrally shallower than in chickens when viewed caudally and laterally (Fig. 5, Meshes S30–52).

Ventral to the cranial roof, the shape of the exoccipital is similar across all four species investigated (Fig. 4, Meshes S30–52). The element is a dorsoventrally deep, curved oval ossification, with its longest axis oriented rostrolaterally. Its caudal end is concave, forming the ventrolateral side of the foramen magnum when viewed caudally. Lateral to the exoccipital, the basioccipital is a dorsoventrally deep element that forms the ventromedial side of the foramen magnum when viewed ventrally. However, it is absent or poorly ossified in some specimens (Meshes S34,35,37,41,42,44, 47–49). In most chicken replicates, the basioccipital has a mediolaterally wide body and narrow rostral and caudal ends when viewed dorsally (Fig. 4, Meshes S31–33). In quails, the body of the basioccipital is mediolaterally narrower than in chickens and is almost uniform in width along its length when viewed ventrally (Fig. 4, Meshes S36,38–40). In Anseriformes, the mallard basioccipital resembles that of chickens (Fig. 4, Meshes S43–45), while the goose basioccipital has a bulbous, mediolaterally wide caudal tip and a gradually tapering rostral tip when viewed dorsally (Meshes S46,50–52).

Ventral to the basioccipital, the basiparasphenoid is a mediolaterally wide sheet of bone that curves dorsally with a trapezoidal profile in ventral view. It fuses with the basisphenoid dorsorostrally, as seen in ventral view (Fig. 4, Meshes S30-52). The shape of the basiparasphenoid appears similar in all four species examined, but with varying degrees of ossification among replicates. Dorsal to the basiparasphenoid, the sellaparasphenoid forms in all four species, but is poorly ossified in some specimens (Meshes S30-32, 36 ,40, 45) and absent in some others (Meshes S34,35,37,38,47). In the specimens where the element is visible, it appears in all four species as a bow-tie-shaped ossification on the caudal side of the sella turcica, in caudomedial view (Meshes S33, 36, 40–44, 46, 48, 50–52).

Lateral to the sellaparasphenoid, the alaparasphenoid is absent in some specimens (Meshes S40,41,47–50) but appears in others as a triangular caudolateral projection arising from the basisphenoid with clade- and species-specific differences, as seen from dorsal view (Meshes S30–39,42–46,51–52). In Galliformes, the alaparasphenoid extends dorsolaterally from the basisphenoid, curving ventrally before terminating caudolaterally, as seen in ventrolateral view (Meshes S30–39). In Anseriformes, the alaparasphenoid extends caudolaterally from the ventrolateral side of the basisphenoid in ventrolateral view (Meshes S42–46,51–52). In Anseriformes, the goose alaparasphenoid is a rostrocaudally short triangular extension of the caudolateral side of the basisphenoid, as seen in dorsal view (Meshes S51–52). Medial to the alaparasphenoid, the basisphenoid appears dorsally as a vertical tube surrounding the sella turcica with a dorsoventrally shallower rostral side in all four species (Meshes S30–52). Rostral to the basisphenoid, the rostroparasphenoid appears, in all species, as a dorsoventrally thick, mediolaterally thin, knife-shaped ossification that tapers rostrally when viewed laterally and exhibits clade and species-specific features. In Anseriformes, the caudal end of the rostroparasphenoid appears mediolaterally wider than in Galliformes, in dorsal view. The goose and quail rostroparasphenoids are proportionally shorter rostrocaudally than those of the mallard and chicken, respectively, in dorsal view (Meshes S30–52).

Ventrolateral to the quadrate, the quadratojugal and jugal appear of similar shape to those in the previous skull stage (Fig. 2, Meshes S30–52). Medial to the jugal, the palatine forms a few species-specific features. In mallards, the vomeral process appears rostrocaudally longer than in geese (Meshes S41–52). Moreover, at the caudoventral end of the palatine, a finger-like projection appears, extending in a dorsocaudal direction from the caudoventral end of the palato-pterygoid articulation surface, seen in caudoventral view, which is absent in geese (Meshes S41–52). However, in Galliformes, the vomeral process appears to be rostrocaudally longer than in the previous skull stage in chickens but is of variable length in quails (Meshes S30–40).

Lateral to the skull, the sclerotic ring appears laterally in all four species, but not in all replicates. It is composed of cubic ossicles that form a planar ring (Fig. 2, Meshes S32–52). The number of ossicles per eye varies, but exhibits considerable overlap among species. In Anseriformes, the number of ossicles varies among mallard replicates between 13 and 15, which is the same range as in geese (Meshes S41–52). In Galliformes, only two chicken specimens exhibited sclerotic rings at this skull stage (Meshes S32–33), whereas in quails 10–15 ossicles appeared (Meshes S34–40).

On the ventrorostral side of the skullmedial to the dentary, the splenial extends rostrally in all four species, with a similar shape to that of the previous skull stage. However, the splenial extends rostroventrally as a concave sheet of bone in Anseriformes; this extension occurs dorsal to the medial side of the caudal ossification of the dentary, as seen in ventromedial view (Meshes S41–52).

Caudal to the splenial, the prearticular appears caudally extended in all four species, but is of largely similar shape to that of the previous skull stage (Meshes S30–52). The caudal extension of the prearticular expands caudomedially in both Anseriformes and Galliformes (Meshes S41–52). Medial to the prearticular, the supraangular appears to be of a similar shape to that of the previous skull stage in all four species (Fig. 4, Meshes S30–52). Ventral to the supraangular, the angular extends rostrally and caudally in all four species, when viewed laterally (Fig. 4, Meshes S30–52). In Galliformes, the caudal extension of the tip of the angular leads to the formation of a mediolaterally narrower caudal tip of the mandible relative to that of the previous skull stage, as seen in ventral view (Fig. 4, Meshes S30–40). Similarly, in Anseriformes the caudal tip of the angular appears mediolaterally narrower than in the previous skull stage, but proportionally longer rostrocaudally in mallards than in geese (Fig. 4, Meshes S41–52).

Ventral to the mandibular elements, the ceratobranchial extends rostrally in all four species, appearing proportionally longest rostrocaudally in mallards, shortest in quails, and of intermediate lengths in geese and chickens, in ventral views (Fig. 4, Meshes S30–52).

### Skull stage 4

This skull stage occurs in chickens at HH stages 40–44 (15–18 days), quails at stage 44 (15–16 days), mallards at stage 41–42 (22–23 days), and geese at stage 42 (26–27 days) (Figs. 2, 3, 4, 5 and Meshes S53–71). Moreover, at this skull stage, the skull approaches its shape at hatching, with a few elements appearing in all four species, including the mesethmoid, epiotic, and opisthotic. Several elements that appear in only some replicates of the previous skull stage appear in all replicates at this skull stage, including the vomer, orbitosphenoid, prootic, and articular.

Caudal to the premaxillary, the frontal process of the nasal extends caudally in a species-specific manner. In Galliformes, the frontal process appears proportionally longer rostrocaudally and mediolaterally wider in chickens than in quails, with a blunt caudal end, in dorsal view (Fig. 3, Meshes S53–63). The mediolaterally wide and blunt caudal end of the frontal process in chickens approximates the shape of its counterpart in geese, in dorsal view. However, in quails, the frontal process is rostrocaudally short and triangular, with a tapering caudal tip in dorsal view. In Anseriformes, the frontal process appears rostrocaudally longer in mallards than in geese, with a tapering caudolateral end in dorsal view (Fig. 3, Meshes S64–71).

Caudal to the lacrimal, the frontal extends medially in all four species when viewed dorsally. Moreover, the frontal’s postorbital side appears dorsoventrally deeper in geese than in most mallard replicates (Fig. 2, Meshes S65–71). Caudal to the frontal, the parietal extends dorsally in all four species in caudolateral view (Fig. 5, Meshes S53–71), with the appearance of the nuchal transverse crest (adapted from cris. nuch. transv. sensu [49]) in mallards and in one goose replicate when viewed caudolaterally (Meshes S64–70).

The epiotic differs slightly between anseriforms and galliforms at this skull stage. In Anseriformes, the epiotic appears as a ventrolaterally extending tube with a dorsoventrally deeper and rostrocaudally narrower rostrolateral end, when viewed caudolaterally (Meshes S64–71). In Galliformes, the epiotic exhibits a similar morphology to the anseriform condition, but its rostrolateral edge extends dorsally, forming a mediolaterally curved canal that laterally faces the squamosal, visible in rostromedial view (Meshes S53–63).

Lateral to the epiotic, the squamosal extends rostro- and dorsomedially in Galliformes and rostrally and caudally in Anseriformes (Fig. 2, Meshes S53–71). The rostromedial extension in galliforms leads to the formation of a mediolaterally wide rostral end of the squamosal, which is rectangular in rostral view. The dorsomedial extension of the galliform squamosal leads to the formation of a wavy parietal-squamosal suture, visible in caudolateral view (Fig. 2, Meshes S53–63). The rostral extension of the anseriform squamosal is most pronounced on its ventral side, which forms the lateral border of the presumptive postorbital process. The caudal extension of the squamosal also leads to the formation of a wavy parietal-squamosal suture, visible in lateral view (Fig. 2, Meshes S64–71). The zygomatic process appears in geese at this skull stage, with a similar shape to that of mallards, when viewed laterally (Fig. 2, Meshes S64–71).

Medial to the squamosal, the prootic appears in all four species as a rostromedially oriented, dorsoventrally deep, wedge-shaped ossification with a rounded caudolateral edge (Meshes S53–68,70). The caudolateral edge of the prootic faces the squamosal (called the squamosal contact (sensu [73]). The proportionally narrow and blunt rostral end of the prootic approaches the basisphenoid when viewed dorsally (Meshes S53–68,70). A few clade-specific features appear at this skull stage, including a rostrocaudally shorter squamosal contact in Anseriformes than in Galliformes. Moreover, two large foramina in the body of the prootic and a dorsoventrally shallow groove containing three small foramina appear in Anseriformes (Meshes S64-68,70). In Galliformes, a singular large foramen appears in the body of the prootic with a dorsoventrally deep groove containing three small foramina (Meshes S53–63). Regarding species-specific features, the basisphenoidal contact is proportionally longer rostrocaudally in chickens than in quails, and the squamosal contact is proportionally shorter rostrocaudally in geese than in mallards, as seen in dorsal view (Meshes S64–68,70).

Ventral to the prootic, the opisthotic appears as a semicircular lateral extension of the exoccipital when viewed ventrolaterally (Meshes S53–68,70). The opisthotic appears on the dorsal side of the exoccipital and is a cup-shaped ossification with deeper medial than lateral sides, and a tapering rostrolateral side, when observed in dorsal view (Meshes S53–68,70). Morphological differences among the four species examined were not discernible. Medial to the opisthotic, the exoccipital is similar in shape across all four species examined (Fig. 4, Meshes S53–71). Medial to the exoccipital, the basioccipital exhibits a similar shape to that of the previous skull stage in all four species (Fig. 4, Meshes S53–71). Rostral to the basioccipital, the basiparasphenoid extends caudally and rostrally, when viewed ventrally, in both anseriforms and galliforms and exhibits some clade-specific features (Fig. 4, Meshes S53–71). The lateral side of the rostral edge of the basiparasphenoid extends in a dorsal direction and fuses with the alaparasphenoid. The medial side of this element extends rostrally, forming a mediolaterally wide and dorsoventrally shallow cavity representing the presumptive Eustachian tube foramen, which is visible from a rostrolateral view (Fig. 4 S53–71). The rostral tip of the basiparasphenoid is blunt in chickens, quails, and geese but pointed in mallards, when viewed rostrolaterally. At the caudal end of the basiparasphenoid, the caudal edge appears wavy in most chicken specimens, and in all quail, mallard, and goose specimens (Meshes S53–55,57–71). It exhibits a finger-like lateral projection in Anseriformes when viewed ventrally (Meshes S64–71).

Rostral to the basiparasphenoid, the sellaparasphenoid exhibits a similar shape to that of the previous skull stage in Galliformes and one goose replicate (Mesh S53-63,71). However, in mallards and other goose replicates, it appears as a rounded ossification with a V-shaped rostromedial edge in caudal view (Meshes S64–70). Lateral to the sellaparasphenoid, the alaparasphenoid extends rostrally and caudally in all four species, and laterally in Anseriformes (Meshes S53–71). In Galliformes, the rostral and caudal extension leads to a rostrocaudally wider alaparasphenoid, relative to that of the previous skull stage, with wavy rostral and caudal edges and a pointed lateral edge when viewed dorsally (Meshes S53–63). In Anseriformes, the alaparasphenoid appears rostrocaudally wider and mediolaterally longer than that in Galliformes in dorsal view, with a similar shape in geese and mallards appearing at this skull stage (Meshes S64–71).

Dorsal to the alaparasphenoid, in all four species examined, the orbitosphenoid appears at this stage and exhibits a more discernible shape in mallards, quails, and chickens than in geese (Fig. 2, Meshes S53–71). The orbitosphenoid appears as a dorsoventrally elongate bony ring surrounding a large foramen, with a mediolaterally wide sheet-like ventral extension, but a mediolaterally narrower dorsal extension, which can be seen in caudolateral view. Laterally, the orbitosphenoid has a dorsocaudally facing concave extension, which is part of the presumptive bony postorbital process, visible in lateral view. In medial view, the dorsomedial and ventromedial extensions of the orbitosphenoid give the medial end of the element a concave appearance. In Galliformes, the foramen in the middle of the element is proportionally smaller than in mallards when viewed medially (Meshes S53–68). Moreover, the presumptive postorbital process appears dorsoventrally shallower and with a more rostral orientation in mallards than in Galliformes (Meshes S53–68). The ventral edge of the orbitosphenoid approaches the sellaparasphenoid with variable morphology. The ventral edge appears wedge-shaped in chickens, but flat in quails (Meshes S53–63). In Anseriformes, the ventral edge appears ventrally planar with a small bifurcation on its medial side (Meshes S64–71). Medioventral to the orbitosphenoid, the basisphenoid and rostroparasphenoid exhibit similar shapes to those of the previous skull stage in all four species (Meshes S53–71).

Lateral to the quadrate, the quadratojugal and jugal appear rostrocaudally longer than in the previous skull stages in all four species.

Lateral to the jugal, the palatine exhibits some clade- and species-specific features on its caudal end (Meshes S53–71). In mallards, the caudomedial projection appears wider, when viewed ventrally, than in the previous skull stage (Meshes S64–71). In Galliformes, the rostrocaudal length of the vomeral process appears similar in chickens and most quail specimens at this skull stage (Meshes S53–71). Moreover, the pterygoid process appears rostrocaudally longer and mediolaterally narrower at its caudal end in chickens than in quails, in dorsal view (Meshes S53–71). Medial to the palatine, the vomer appears at this skull stage with clade- and species-specific features (Fig. 4, Meshes S53–71). In Galliformes, the vomer appears as a small oval-shaped ossification with a rostrocaudally oriented long axis and is more rostrally positioned in quails relative to chickens, as seen in ventral view (Fig. 4, Meshes S53–63). Moreover, in some chicken replicates, the vomer appears caudally bifurcated in ventral view (Meshes S53,55,56,58,60). On the other hand, in anseriforms, the vomer appears as a dorsoventrally deep wedge-shaped ossification that forms medial to the vomeral process of the palatine (Fig. 2, Meshes S64–71). Moreover, on the dorsorostral side of the vomer, a process extends towards the intermaxillary region, which is rostrocaudally longer in mallards than in geese, when viewed ventrolaterally (Meshes S64–71). On the caudal side of the vomer, the element decreases in dorsoventral depth, forming a sheet-like extension ventral to the rostroparasphenoid; this extension is rostrocaudally longer in mallards than in geese, viewed ventrolaterally (Meshes S64–71).

Dorsal to the vomer, the mesethmoid appears as a sheet-like medial ossification with clade- and species-specific features. In Anseriformes, the mesethmoid appears as a dorsoventrally vertical sheet that is tilted dorsolaterally in mallards and ventrolaterally in geese, when viewed caudally (Fig. 2, Meshes S65–71). In Galliformes, it has a dorsoventral orientation that curves laterally on the dorsal side of the element, when viewed caudally (Fig. 2, Meshes S53–63). Lateral to the mesethmoid, the sclerotic ring is similar to that of the previous skull stage, with overlapping ranges of ossicle number in the four species: 13–14 in chickens and quails, 14–15 in mallards, and 13–15 in geese (Fig. 2, Meshes S53–71).

Medial to the dentary, the splenial also appears of a similar shape to the previous skull stage in all four species (Meshes S53–71). However, in chickens, a short dorsorostral process appears, bifurcating the rostral edge of the splenial, visible in medial view (Meshes S53–56). Caudal to the splenial, the prearticular exhibits a similar shape to that of the previous skull stages in all four species (Meshes S53–71). Caudal to the prearticular, the articular appears, caudally, in all four species as a mediolaterally compressed dorsoventrally vertical cone-shaped ossification, with some clade- and species-specific features (Fig. 4, Meshes S53–71). In Anseriformes, the caudal side has a mediolaterally wide ventrolaterally convex surface that is absent in Galliformes. In Galliformes, the articular forms without an apparent suture with the prearticular in chickens, unlike in quails, visible in caudoventral view (Meshes S53–63).

Lateral to the articular, the supraangular exhibits a similar shape to that of the previous skull stage in all four species (Fig. 2, Meshes S53–71). However, in chickens a short rostroventral process appears, in lateral view, which forms the caudoventral boundary of the mandibular fenestra (Meshes S53–60). Ventral to the supraangular, the angular exhibits a similar shape to that of the previous skull stage in all four species (Fig. 4, Meshes S53–71). Ventral to the mandible, the ceratobranchial appears slightly rostrocaudally elongated relative to previous skull stages in all four species (Fig. 4, Meshes S53–71).

### Skull stage 5

The final embryonic skull stage occurs at the hatching stage. In chickens, this occurs at HH stage 45 (21 days), quails at stage 45 (16.5 days), mallards at stage 43 (26 days), and geese at stage 42 (28 days) (Figs. 2, 3, 4, 5 and Meshes S72–84). All the cranial elements have ossified by this skull stage, with the notable absence of the retroarticular, postorbital, and basipterygoid processes, along with the mandibular condyles of the quadrate (Meshes S72-84).

In the rostrum, the beaks of galliforms are rostrocaudally shorter and dorsoventrally deeper than those of anseriforms, with a pointed rostral tip. By contrast, those of the anseriforms have rostrocaudally elongate, dorsoventrally flat, and mediolaterally wider bills. In chickens, the beak is more ventrally curved than in quails, and goose beaks are rostrocaudally shorter, mediolaterally narrower, and dorsoventrally deeper than those of mallards (Fig. 2). Caudal to the beak, the orbital region appears proportionally larger in Galliformes than in Anseriformes, in lateral view. Moreover, the supraorbital region is more dorsally concave in chickens than in quails when viewed laterally (Fig. 2). Caudal to the orbit, the braincase is dorsoventrally deeper and rostrocaudally shorter in Anseriformes than in Galliformes. In Galliformes, the chicken braincase is mediolaterally narrower and dorsoventrally deeper than in quails (Figs. 2, 5). Ventral to the braincase, a galliform synapomorphy becomes clearly discernible: the presence of a rostromedial foramen on the otic process of the quadrate, which is visible in caudomedial view (Table 2, Chr.52.1; Meshes S72–77). Ventral to the quadrate, the caudal end of the mandible is mediolaterally wider in Galliformes than in Anseriformes, while the rostral tip of the mandible is pointed in Galliformes, as seen in ventral view. Moreover, in Anseriformes, the caudal end of the mandible is mediolaterally wider in geese than in mallards, as seen in ventral view (Fig. 4).

## Data Availability

All meshes were uploaded to morphosource database (https://www.morphosource.org/projects/000717257?utf8=%E2%9C%93&per_page=100&locale=en&view=gallery).

## Appendix

**Table 1:** List of specimens used for comparing skull osteogenesis in four species: chicken (*Gallus gallus*)*,* Japanese quail (*Coturnix japonica*)*,* mallard (*Anas platyrhynchos*)*,* and swan goose (*Anser cygnoides*)

Numbers	Name	Species	Stage	Age	Replicate number	Skull stage	Morphosource code
S1	Stage 1_HH35_R.1	*G. gallus*	HH35	8–9 days	1	1	GG35R1
S2	Stage 1_HH35_R.2	*G. gallus*	HH35	8–9 days	2	1	GG35R2
S3	Stage 1_HH36_R.3	*G. gallus*	HH36	10 days	3	1	GG36R1
S4	Stage 1_HH36_R.4	*G. gallus*	HH36	10 days	4	1	GG36R2
S5	Stage 1_HH36_R.5	*G. gallus*	HH36	10 days	5	1	GG36R3
S6	Stage 1_HH36_R.6	*G. gallus*	HH36	10 days	6	1	GG36R4
S7	Stage 1_35_R.1	*C. japonica*	35	8–8.5 days	1	1	CJ35R1
S8	Stage 1_35.5_R.2	*C. japonica*	35.5	8–8.5 days	2	1	CJ35.5R1
S9	Stage 1_35.5_R.3	*C. japonica*	35.5	8–8.5 days	3	1	CJ35.5R2
S10	Stage 1_36_R.1	*A. platyrhynchos*	36	11.5–12.5 days	1	1	AP36R1
S11	Stage 1_37_R.2	*A. platyrhynchos*	37	12.5–13 days	2	1	AP37R1
S12	Stage 1_37_R.3	*A. platyrhynchos*	37	12.5–13 days	3	1	AP37R3
S13	Stage 1_36_R.1	*A. cygnoides*	36	14 days	1	1	AC36R1
S14	Stage 2_HH37_R.1	*G. gallus*	HH37	11 days	1	2	GG37R1
S15	Stage 2_HH37_R.2	*G. gallus*	HH37	11 days	2	2	GG37R4
S16	Stage 2_HH38_R.3	*G. gallus*	HH38	12 days	3	2	GG38R1
S17	Stage 2_HH38_R.4	*G. gallus*	HH38	12 days	4	2	GG38R2
S18	Stage 2_HH38_R.5	*G. gallus*	HH38	12 days	5	2	GG38R3
S19	Stage 2_37_R.1	*C. japonica*	37	9.5 days	1	2	CJ37R1
S20	Stage 2_37_R.2	*C. japonica*	37	9.5 days	2	2	CJ37R2
S21	Stage 2_38.5_R.3	*C. japonica*	38.5	10 days	3	2	CJ38.5R1
S22	Stage 2_39_R.4	*C. japonica*	39	10.5 days	4	2	CJ39R1
S23	Stage 2_37.5_R.1	*A. platyrhynchos*	37.5	13.5 days	1	2	AP37.5R1
S24	Stage 2_38_R.2	*A. platyrhynchos*	38	14–15 days	2	2	AP38R1
S25	Stage 2_38_R.3	*A. platyrhynchos*	38	14–15 days	3	2	AP38R2
S26	Stage 2_38_R.4	*A. platyrhynchos*	38	14–15 days	4	2	AP38R3
S27	Stage 2_36_R.1	*A. cygnoides*	36	12 days	1	2	AC36R1
S28	Stage 2_37_R.2	*A. cygnoides*	37	15–16 days	2	2	AC37R1
S29	Stage 2_37_R.3	*A. cygnoides*	37	15–16 days	3	2	AC37R3
S30	Stage 3_HH39_R.1	*G. gallus*	HH39	13 days	1	3	GG39R1
S31	Stage 3_HH39_R.2	*G. gallus*	HH39	13 days	2	3	GG39R2
S32	Stage 3_HH39_R.3	*G. gallus*	HH39	13 days	3	3	GG39R3
S33	Stage 3_HH40_R.4	*G. gallus*	HH40	14 days	4	3	GG40R1
S34	Stage 3_40_R.1	*C. japonica*	40	11 days	1	3	CJ40R1
S35	Stage 3_40_R.2	*C. japonica*	40	11 days	2	3	CJ40R2
S36	Stage 3_42_R.3	*C. japonica*	42	12–13 days	3	3	CJ42R1
S37	Stage 3_42_R.4	*C. japonica*	42	12–13 days	4	3	CJ42R2
S38	Stage 3_42_R.5	*C. japonica*	42	12–13 days	5	3	CJ42R3
S39	Stage 3_43_R.6	*C. japonica*	43	14 days	6	3	CJ43R1
S40	Stage 3_43_R.7	*C. japonica*	43	14 days	7	3	CJ43R2
S41	Stage 3_39_R.1	*A. platyrhynchos*	39	16 days	1	3	AP39R1
S42	Stage 3_40_R.2	*A. platyrhynchos*	40	18 days	2	3	AP18R1
S43	Stage 3_40_R.3	*A. platyrhynchos*	40	19 days	3	3	AP19R1
S44	Stage 3_40_R.4	*A. platyrhynchos*	40	19 days	4	3	AP19R2
S45	Stage 3_41_R.5	*A. platyrhynchos*	41	20 days	5	3	AP20R1
S46	Stage 3_37_R.1	*A. cygnoides*	37	15–16 days	1	3	AC37R2
S47	Stage 3_37_R.2	*A. cygnoides*	37	15	2	3	AC40-41?R2
S48	Stage 3_38_R.3	*A. cygnoides*	38	16	3	3	AC38R1
S49	Stage 3_39_R.4	*A. cygnoides*	39	18	4	3	AC39R1
S50	Stage 3_40_R.5	*A. cygnoides*	40	~ 19 days	5	3	AC40-41?R3
S51	Stage 3_40_R.6	*A. cygnoides*	40	19	6	3	AC4019R1
S52	Stage 3_41_R.7	*A. cygnoides*	41	20	7	3	AC4120R1
S53	Stage 4_HH40_R.2	*G. gallus*	HH40	14 days	1	4	GG40R2
S54	Stage 4_HH41_R.3	*G. gallus*	HH41	15 days	2	4	GG41R3
S55	Stage 4_HH41_R.4	*G. gallus*	HH41	15 days	3	4	GG41R4
S56	Stage 4_HH41_R.5	*G. gallus*	HH41	15 days	4	4	GG41R5
S57	Stage 4_HH41_R.6	*G. gallus*	HH41	15 days	5	4	GG41R3
S58	Stage 4_HH42_R.7	*G. gallus*	HH42	16 days	6	4	GG42R1
S59	Stage 4_HH43_R.8	*G. gallus*	HH43	17 days	7	4	GG43R1
S60	Stage 4_HH44_R.9	*G. gallus*	HH44	18 days	8	4	GG44R1
S61	Stage 4_44_R.1	*C. japonica*	44	15–16 days	1	4	CJ44R1
S62	Stage 4_44_R.2	*C. japonica*	44	15–16 days	2	4	CJ44R2
S63	Stage 4_44_R.3	*C. japonica*	44	15–16 days	3	4	CJ44R3
S64	Stage 4_41_R.6	*A. platyrhynchos*	41	21 days	1	4	AP21R1
S65	Stage 4_41_R.7	*A. platyrhynchos*	41	21 days	2	4	AP21R2
S66	Stage 4_42_R.1	*A. platyrhynchos*	42	22 days	3	4	AP22R1
S67	Stage 4_42_R.2	*A. platyrhynchos*	42	22 days	4	4	AP23R1
S68	Stage 4_42_R.3	*A. platyrhynchos*	42	24 days	5	4	AP24R1
S69	Stage 4_42_R.1	*A. cygnoides*	42	22 days	2	4	AC42R1
S70	Stage 4_41-42_R.2	*A. cygnoides*	41–42	~ 22 days	3	4	AC40-41?R2
S71	Stage 4_42-42.5_R.3	*A. cygnoides*	42–42.5	~ 23 days	4	4	AC42-43?R1
S72	Stage 5_HH45_R.1	*G. gallus*	HH45	25 days	1	5	GG46R1
S73	Stage 5_HH45_R.2	*G. gallus*	HH45	25 days	2	5	GG46R2
S74	Stage 5_HH45_R.3	*G. gallus*	HH45	25 days	3	5	GG46R3
S75	Stage 5_45_R.1	*C. japonica*	45	16.5 days	1	5	CJ45R1
S76	Stage 5_45_R.2	*C. japonica*	45	16.5 days	2	5	CJ45R2
S77	Stage 5_45_R.3	*C. japonica*	45	16.5 days	3	5	CJ45R3
S78	Stage 5_43_R.1	*A. platyrhynchos*	43	25 days	1	5	AP25R1
S79	Stage 5_43_R.2	*A. platyrhynchos*	43	25 days	2	5	AP25R2
S80	Stage 5_43_R.3	*A. platyrhynchos*	43	25 days	3	5	AP25R3
S81	Stage 5_43_R.4	*A. platyrhynchos*	43	25 days	4	5	AP26R1
S82	Stage 5_44_R.1	*A. cygnoides*	43	26 days	1	5	AC44R1
S83	Stage 5_43_R.2	*A. cygnoides*	43	~ 25 days	2	5	AC45R3
S84	Stage 5_43_R.3	*A. cygnoides*	43	~ 25 days	3	5	AC45R4

**Table 2:** List of Galloanserae, Galliformes and Anseriformes cranial synapomorphies inferred from extant taxa in the phylogenetic dataset of [32]. The osteological synapomorphies present during skull formation stages are shown in bold. *This synapomorphy could not be determined because of lack of myological data of Anseriform embryos. † This synapomorphy does not appear in chickens or quails

Clade	Character No	Description	Timing of appearance
Galloanserae	1.2	Premaxilla less than 50% total cranium length	Pre-hatching
	12.1	Orbital process of lacrimal dorsoventrally short	Pre-hatching
	20.1	Tympanic recess bound ventrally by parasphenoid wing	Post-hatching
	68.1	Medial process of mandible long, narrow, and dorsally oriented	Post-hatching
	292.2	Retroarticular process hooked	Post-hatching
Anseriformes	6.1	Lamellae present in upper beak	Pre-hatching
	7.1	Maxillopalatine processes of premaxilla fused medially, caudally enclosing elongate ventromedial fenestra	Post-hatching
	19.1	Impression of pars coronoidea of m. adductoris mandibulae externus restricted to medial surface of postorbital process (instead of present laterally on cranium)	Unknown*
	25.1	Basilar tubercles (mamillar tuberosities) on medial parasphenoid process large	Post-hatching
	36.1	Palatines completely fused along midline	Post-hatching
	37.1	Caudal end of vomer fused	Post-hatching
	38.1	Caudal end of vomer does not wrap around parasphenoid rostrum	Post-hatching
	48.1	Submeatic prominence on caudomedial surface of lateral process on quadrate	Post-hatching
	51.1	Caudomedial foramen on otic process of quadrate	Pre-hatching
	53.1	Prominent orbital crest on orbital process of quadrate	Post-hatching
	58.0	Dorsolateral margin extending from otic process to dorsal tip of orbital crest on quadrate straight or only slightly concave	Post-hatching
	295.1	Suture between nasal and premaxilla obliterated at tomial margin	Post-hatching
Galliformes	31.1	Vagus foramen caudal or mesial to paroccipital notch	Post-hatching
	43.1	Articulation between pterygoid and palatine is a ball and socket joint with one main condyle	Post-hatching
	52.1	Rostromedial foramen on otic process of quadrate	Pre-hatching
	54.1	Basiorbital foramen on otic process of quadrate	N/A†
